# Rising Environmental Inequalities and Their Relationship to Racial and Socioeconomic Disparities in the US Southwest

**DOI:** 10.1021/acs.est.4c14369

**Published:** 2025-08-13

**Authors:** Yuanhui Zhu, Soe W. Myint, Danica Schaffer-Smith, Daoqin Tong, Yuyu Zhou, Yubin Li, Rebecca L. Muenich

**Affiliations:** † Department of Geography and Environmental Studies, 7174 Texas State University, San Marcos, Texas 78666, United States; ‡ The Meadows Center for Water and the Environment, San Marcos, Texas 78666, United States; § US Fish and Wildlife Service, Raleigh, North Carolina 27636, United States; ∥ School of Geographical Sciences and Urban Planning, 7864 Arizona State University, Tempe, Arizona 85281, United States; ⊥ Department of Geography, 366142 The University of Hong Kong, Hong Kong 999077, China; # Institute for Climate and Carbon Neutrality, The University of Hong Kong, Hong Kong 999077, China; ¶ Department of Biological and Agricultural Engineering, 3341 University of Arkansas, Fayetteville, Arkansas 72701, United States

**Keywords:** race/ethnicity, social-environmental disparities, inequality changes, drought, urban warming, water use, climate impact

## Abstract

The US Southwest, the hottest and driest region in the US, faces significant climate-coupled challenges to sustainable development by human and natural systems. This study examines how inequalities in race and ethnicity have shifted with climate impact, analyzing disparities in socioeconomic conditions, remote sensing-based environmental metrics, and environmental changes (2000–2020). Results reveal widespread racial and ethnic disparities in social and environmental conditions. From 2000 to 2020, environmental inequalities increased, with significant differences in land surface temperature (LST) (Sen’s slope differences: 0.00620 °C/year for Hispanic vs non-Hispanic (ethnic group); 0.00366 °C/year for people of color vs non-Hispanic White (racial group) and actual evapotranspiration (ETa) as a proxy for consumptive water use (−0.00167 mm/year for ethnic groups). LST disparities among races/ethnicities grew in warmer regions, while they decreased in temperate and cold areas. Water resource disparities also increased across different ethnic groups in drier neighborhoods, with a slope of ETa differences at −0.00237 mm/year. These findings highlight the linkages between environmental inequality, physiography, and climate change, demonstrating that climate change exacerbates these disparities. We recommend that urban planners and managers expand green spaces in vulnerable neighborhoods and communities to mitigate the disproportionate impact of climate change and enhance urban resilience to heat extremes and droughts for the entire urban setting.

## Graphical abstract

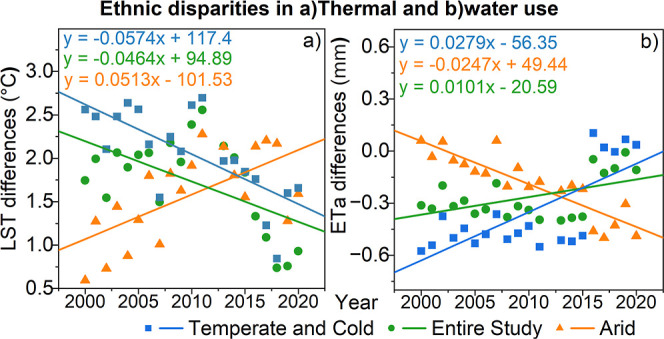

## 1 Introduction

Social and environmental inequities pose significant challenges for the US, as this phenomenon commonly contributes to various public security and health issues.^1,2^ Vulnerable groups, including marginalized communities and racial/ethnic minorities, face heightened risks from heat- and drought-related mortality and morbidity.^1,3–9^ Built environment characteristics, such as access to green space, transportation network, open land, and building density, exacerbate disparities in heat exposure and associated water consumption (e.g., through irrigation and evapotranspiration), emphasize the trade-offs between urban cooling benefits and water sustainability challenges, and underscore the need to address these inequities for sustainable urban development.^7,10–14^

Environmental inequality refers to the disproportionate burden of environmental exposures (e.g., heat, drought, pollution) on marginalized communities.^15^ Existing research has documented widespread social and environmental inequality problems, indicating that Hispanic, people of color, and other minority communities experience higher urban heat exposure and lower vegetation coverage.^3,5,16^ Disadvantaged groups, defined by social, economic, or environmental marginalization, are disproportionately exposed to heat and its related health risks.^17–19^ Poorer neighborhoods typically experience higher land surface temperatures (LST) than wealthier ones, further exacerbating inequities.^3^ Most studies have focused on single metrics, such as urban heat island or green space, or static data from specific years.^20,21^ However, the socioeconomic and environmental factors driving inequalities are complex, dynamic, and interactive, involving intersecting influences such as historical segregation and redlining, differential land use patterns, variations in socioeconomic status, climate variability, and differences in community adaptive capacity.^2,3,5,7,22,23^ These overlapping factors make static analyses potentially inconclusive. There is also little knowledge regarding changes in environmental inequalities over time, especially in the context of accelerating global environmental change.^1,20^ Disparities in evaporative stress index (ESI, as a proxy for drought) and actual evapotranspiration (ETa, as a proxy for consumptive water use) over larger spatial scales at fine resolution have not been explored.

The US Southwest, characterized as the hottest and driest region in the US, is particularly vulnerable to these issues.^10,14^ The 21st-century drought has intensified these challenges, with Colorado, Utah, Arizona, southern Nevada, and southern California experiencing below-average precipitation since 2000.^1,24,25^ More than half of the region is considered “Arid” according to the Köppen-Geiger climate classification.^26^ As climate change brings hotter and drier conditions, water availability is increasingly limited, raising concerns about the inequitable distribution of drought impacts across racial/ethnic groups.^1,3,10,27^ However, we know little about the differences in drought and water availability for different racial/ethnic communities in the region under global climate change.

This study addresses these gaps by exploring social and environmental inequalities in 46 major US Southwest cities. We integrate high-resolution ECOSTRESS (ECOsystem Spaceborne Thermal Radiometer Experiment on Space Station) data (LST, ESI, and ETa) with US Census sociodemographic parameters at the block group level. Using Landsat-based LST and ETa from 2000 to 2020, we analyze disparities among racial/ethnic groups over time in light of global climate changes. The specific objectives are to (i) examine the disparities in socioeconomic conditions (including median household income, per capita income, poverty status, and property values) and environmental changes among races/ethnicities, (ii) dynamically assess inequality changes over time, (iii) evaluate the impact of climate changes on environmental inequalities, and (iv) propose equitable policies for sustainable urban planning. By focusing on summer months, when heat and water scarcity impacts are most severe,^5,28^ this study provides actionable insights into mitigating environmental inequalities and improving resilience in vulnerable communities.

## 2 Materials and Methods

### 2.1 Study Area

The Southwestern US is a region of the western US that is warmer and drier than other areas of the US. The definition of the Southwestern US varies, but Arizona (AZ) and New Mexico (NM) are always included. In addition to these two states, our study area contained all possible Southwestern areas, including California (CA), Colorado (CO), Kansas (KS), Nevada (NV), Oklahoma (OK), Texas (TX), and Utah (UT) (Figure S1). The boundaries of the 46 largest urban areas defined by the US Census Bureau from these nine states were selected (https://www.census.gov/geographies/mapping-files/2018/geo/carto-boundary-file.html) that contained more than 250,000 residents. The list of cities is provided in Table S1 of the Supporting Information. We also identified the Köppen-Geiger climate zones for each area to explore whether environmental inequality varies across different climate zones. There are three climate zone categories—arid (B), temperate (C), and cold (D), and eight subclimates in our study area.

### 2.2 Data Acquisition & Processing

We analyzed demographic and socioeconomic data, ECOSTRESS products, and Landsat-based environmental change data sets (LST and ETa). A detailed description of the data sets is shown in Table S2. Racial/ethnic demographic and socioeconomic data at the block level were sourced from the US Census Bureau. The demographic data include categories such as people of color, non-Hispanic White, White alone, Black or African American alone, American Indian and Alaska Native alone, Asian alone, Native Hawaiian and Other Pacific Islander alone. In contrast, ethnic groups refer to individuals identifying as Hispanic versus non-Hispanic. Socioeconomic metrics (median household income, per capita income, poverty status, and property values) were summarized from ACS 5 year estimates. All these data were downloaded as continuous variables and linked to the corresponding block groups.

We used a time-segmented approach to better capture population dynamics across the 2000–2020 study period. These years were chosen because they are based on decennial Census counts conducted with the highest accuracy and consistency by the U.S. Census Bureau. Specifically, demographic data from the 2000 Census were used to represent the years 2000–2005, 2010 Census data were used for 2006–2015, and 2020 Census data were used for 2016–2020. Socioeconomic metrics (median household income, per capita income, poverty status, and property values) were summarized from ACS 5 year estimates. The percentage of households below the poverty level was calculated as the proportion of households with incomes below the poverty threshold in each block group. LST, reflecting urban warming effects, indicates heat exposure and stress.^24^ ESI highlights drought conditions by identifying vegetation stress due to water shortages.^14^ ETa represents consumptive water use, including evaporation, transpiration, and human consumption, which does not return to water systems.^29^ These metrics provide insights into human water use and its hydrologic impacts.

#### 2.2.1 Demographic and Socioeconomic Data

We analyzed all major Southwestern US urban areas at the Census block group level. All socio-economic data were obtained from the US Census Bureau. To explore the environmental inequality across communities with a range of racial composition and diversity, we compiled other demographic information from the Census at the block group level. Race from the US Census includes six categories—White alone (WHI), Black or African American alone (BLA), American Indian and Alaska Native alone (AIA), Asian alone (ASI), Native Hawaiian and Other Pacific Islander alone (NHO), and two or more races (TMR). Ethnicity is associated with two categories—Hispanic (HIS) and non-Hispanic (NHIS). We also create a People of Color category—including all Hispanic populations and all populations that do not identify as White alone, and its complementary set—non-Hispanic White (NHW).^5^

Recognizing that the assessment of social-environmental inequalities should compare different races/ethnicities to explore their differences, we mainly considered two contrary groups of races/ethnicities—Hispanic versus non-Hispanic (referred to as the ethnic groups) and people of color versus non-Hispanic White (referred to as racial groups)—to evaluate their inequality metrics.^5^

#### 2.2.2 ECOSTRESS Data

We acquired NASA’s ECOsystem Spaceborne Thermal Radiometer Experiment on Space Station data sets for short ECOSTRESS data sets, including level-2 (L2) land surface temperature (LST), level-3 (L3) actual evapotranspiration (ETa), and level-4 (L4) evaporative stress index (ESI).^29^ Only the summer season (June, July, and August) of 2020 data were synthesized to explore the relationship between the percentage of racial/ethnic population and environmental variables (LST, ETa, and ESI). We chose at least three scenes for each urbanized area for summer daytime (11:00–16:00 local solar time) LST to reflect the mean values of summer daytime temperature.^10^ ETa and ESI products generally have less available data for urbanized areas because of the high surface heterogeneity of urban landscapes. Consequently, we employed all available data sets during the summer and calculated their arithmetic averages for ETa and ESI to ensure comprehensive coverage of entire urbanized areas. We assigned the mean values of ECOSTRESS data to each boundary of the US Census block groups. Because our analysis emphasizes within-city comparisons across cities, the effect of variable overpass timing is further minimized, since neighborhoods within each city are observed under relatively similar diurnal conditions.

#### 2.2.3 Environmental Change Database

The Environmental Change Database was processed using the Google Earth Engine (GEE) platform. We developed the statistical mono-window (SMW) and the operational version of the simplified surface energy balance (SSEBop) algorithm in the GEE platform to compute Landsat-based LST and ETa, respectively.^30–34^ Consequently, data sets for LST and ETa with a 30m resolution spanning from 2000 to 2020 were generated. All available Landsat images with cloud cover lower than 75% in the summer (June, July, and August) from 2000 to 2020 were obtained based on the GEE platform to analyze LST and ETa changes. The scan line corrector (SLC)-off no-data stripes from individual landsat-7 scenes were eliminated. There are a total of 8860 scenes (including landsat 4, 5, 7, and 8) to cover our study area from 2000 to 2020 fully. While landsat data are acquired with a consistent overpass time, approximately 10:00 a.m. local time, the influence of geographic variation in solar angle and sunrise time across may affect absolute LST values. To mitigate this limitation, our primary analysis focused on within-city comparisons and long-term temporal trends, thus reducing bias introduced by geographic or diurnal radiative differences.

All scenes removed contaminated pixels (e.g., clouds and cloud shadows) based on landsat quality assessment (QA) band. Finally, the Mann-Kendall (MK) trend analysis was employed to derive Sen’s slope for LST and ETa, spanning from 2000 to 2020, facilitating an examination of environmental changes. We assigned the mean values of environmental change variables to each boundary of the US Census block groups. More details about the algorithm to retrieve LST and ETa can be seen in Supporting Information (S.1) and Landsat-based environmental change (2000–2020) metrics over the state capitals within our study areas are shown in Figures S2–S6.

### 2.3 Data Analysis

This study analyzed social-environmental inequalities across 46 major urban areas in the U.S. Southwest using block group-level data. A flowchart that demonstrates the analytical procedure is presented in Figure S7. Two complementary approaches were employed: population-weighted mean and dominant group-based analysis. The population-weighted mean method uses the population size of each racial/ethnic group within block groups as the weight, though it may be skewed by high population concentrations.^5^ In contrast, the dominant group-based method calculates environmental metrics for block groups where a single racial/ethnic group is predominant, minimizing the impact of extreme population sizes. However, this approach may be biased by variations in block group area (typically 1–5 km^2^) and the uneven spatial distribution of demographic and environmental factors across climate zones.^35^ Comparing both methods can assess the robustness of the results. Further methodological details are provided below.

#### 2.3.1 Global Weighted Means to Explore Widespread Inequality

To assess widespread inequalities, the average values of all variables (social-economic, environmental, and environmental change data sets) were calculated as weighted means across the study area. The weights, based on the population of specific racial/ethnic groups in corresponding block groups, were used to compute global weighted means (eq 1).^5^ These means were compared across racial/ethnic groups to evaluate inequalities and correlations between racial/ethnic percentages and given variables were also quantified.

1 $$Global\;weight\;mean=\frac{\sum_{i=1}^{n}w_{i}x_{i}}{\sum_{i=1}^{n}w_{i}}$$

where *x*_*i*_ represents the mean value of the specific variable (e.g., ET, LST) for the *i*-th block group; *w*_*i*_ represents the weight for the *i*-th block group (in this case, the population of a specific race/ethnicity, such as the population of White alone, in the *i*-th block group); *n* is the total number of block groups (43,388 block groups in this case) within the entire study areas.

#### 2.3.2 Interurban Weighted Means for Identifying Statistical Differences

To identify whether statistically significant differences existed in given variables between contrary groups (ethnic groups: People of color vs non-Hispanic White and racial groups: Hispanic vs non-Hispanic), we calculated the weighted means of all given variables in each urban area, referred to as interurban weighted means. The interurban weights correspond to the population of a specific race/ethnicity residing in a block group within each urban area, differing from the global weighted means of the given variables across the entire study area. Weighted means for 46 cities in the US Southwest were compared to test statistical differences between groups.

The Wilcoxon nonparametric test was used to assess significant differences in variables between racial and ethnic groups. This test does not assume normal data distribution and is well-suited for paired observations.^36^ In this study, interurban weighted means for races (People of color vs non-Hispanic White) or ethnicities (Hispanic vs non-Hispanic) were treated as paired observations to test differences.

#### 2.3.3 Inequality Changes between 2000 and 2020 within Climate Zones

To better understand environmental inequalities, we analyzed disparities between contrary groups within distinct climate zones based on the Köppen-Geiger classification.^26^ Regions were divided into two groups—Arid (24 of 46 cities) and temperate and cold (22 cities)—to account for variations in temperature and drought patterns. Within each climate group, we conducted two analyses: (a) statistical comparisons of interurban weighted means for environmental and environmental change variables using the Wilcoxon test, and (b) assessments of inequality shifts by analyzing changes in land surface temperature (LST) and evapotranspiration (ETa) between 2000 and 2020.

To assess inequality shifts and minimize bias from neighborhoods with exceptionally large population concentrations based on the weighted-mean method, we examined LST and ETa trends between contrary groups (Hispanic vs non-Hispanic and People of color vs non-Hispanic White) across neighborhoods dominated by specific racial/ethnic groups in each city (intraurban approach). Dominant-group neighborhoods were defined using city-specific percentiles: (1) Hispanic-dominated (Hispanic population > 75th percentile per city), (2) non-Hispanic-dominated (Hispanic population < 25th percentile per city), (3) ethnically diverse (Hispanic population between 25th and 75th percentiles per city. The same criteria were applied for identifying non-Hispanic White- and people of color-dominated areas. City-based percentiles (e.g., >75th percentile) can be expected to evaluate racial/ethnic dominance relative to each city’s demographic context and reflect local inequality patterns, which is essential given the wide variation in racial/ethnic compositions across our 46 study cities. Annual mean LST and ETa values were then calculated from 2000 to 2020 for each dominant-group polygon.

This intraurban approach accounts for the substantial environmental variability across the 46 study cities, which span diverse climate zones and land cover types, and avoids confounding effects introduced by comparing cities with inherently different baseline conditions.^5^ For example, comparing LST values between Phoenix (hot and dry) and San Francisco (cool and humid) would obscure sociodemographic influences due to background climatic differences. Moreover, cities with extreme population dominance (e.g., Laredo, TX, with 95.22% Hispanic in 2020) were excluded to reduce skewed results. In connection to this concern, an additional information is provided in the Supporting Information (Table S3). We retain at least 70% of the population for each racial/ethnic group after applying the exclusion criteria to make sure our data remain representative (Table S4).

#### 2.3.4 LST and ETa Differences between the Contrary Groups from 2000 to 2020

Trends in environmental inequality changes cannot be assessed by the differences in mean LST and ETa Sen’s slope among different races/ethnicities in the 21 years of the study period. The differences in environmental metrics (LST and ETa) among contrary groups were assessed between 2000 and 2020 to analyze changing trends of environmental inequalities. Annual mean LST and ETa values were first computed based on the polygon boundaries of neighborhoods dominated by two contrary groups of races/ethnicities. Then, their differences for each year were calculated using the Hispanic-dominated and non-Hispanic White-dominated neighborhoods or people of color-dominated and non-Hispanic White-dominated neighborhoods. Finally, the trends in LST and ETa differences between these contrary groups were derived from the calculated differences spanning the years 2000 to 2020.

## 3 Results and Discussion

### 3.1 Widespread Inequality in Social-Environmental Conditions by Races/Ethnicities Across Major US Southwest Urban Areas

The global weighted means of social-environmental metrics by race/ethnicity across major US Southwest cities is shown in Table 1. With respect to race, socioeconomic outcomes in 2020 have remained consistently better for White and Asian residents compared to other racial minorities. These minorities had similar poor outcomes for socioeconomic variables. The percentage of Whites and Asians at the block group level had a significant positive association with positive socioeconomic indicators—median household income, per capita income, and median household property values—and a negative association with indicators of socioeconomic distress—namely, the percentage of households below the poverty level. Overall, all positive socioeconomic indicators decreased as the percentage of other minority groups increased, and vice versa.

**Table 1:** Global Weighted Means of Social-Environmental Metrics by Races Across Major US Southwest Cities^a^

								contrary groups			
			race					ethnic groups		racial groups	
variables		mean ± SD	WHI	BLA	AIA	ASI	NHO	HIS	NHIS	PCOL	NHISW
socioeconomic conditions in 2020	^+^MHI (K$)	78.09 ± 0.48	79.88	64.74	64.75	80.96	68.62	64.63	79.68	68.31	82.71
			(0.38**)	(−0.28**)	(−0.31**)	(0.32**)	(−0.06**)	(−0.51**)	(0.51**)	(−0.44**)	(0.44**)
	^+^PCI (K$)	37.01 ± 0.27	37.25	28.68	28.11	35.97	29.99	27.66	36.81	29.71	38.96
			(0.5**)	(−0.24**)	(−0.31**)	(0.21**)	(−0.11**)	(−0.59**)	(0.59**)	(−0.56**)	(0.56**)
	^–^PHBPL	0.13 ± 0.0014	0.12	0.17	0.16	0.12	0.15	0.16	0.12	0.15	0.11
			(−0.41**)	(0.26**)	(0.24**)	(−0.16**)	(−0.01)	(0.45**)	(−0.45**)	(0.45**)	(−0.45**)
	^+^MHPV (K$)	430.15 ± 3.93	363.12	294.62	285.16	358.77	304.01	283.47	360.77	303.82	376.2
			(0.12**)	(−0.23**)	(−0.25**)	(0.47**)	(−0.03**)	(−0.38**)	(0.38**)	(−0.21**)	(0.21**)
state of environment in 2020	^–d^aytime LST (deg)	43.57 ± 0.06	43.6	44.25	44.25	43.9	44.33	44.3	43.68	44.18	43.51
			(−0.13**)	0	(0.15**)	(−0.16**)	(0.04**)	(0.27**)	(−0.27**)	(0.17**)	(−0.17**)
	^+^ESI	0.4697 ± 0.0023	0.46	0.447	0.449	0.449	0.448	0.448	0.458	0.449	0.462
			(0.08**)	(0.25**)	(−0.12**)	(−0.15**)	(−0.22**)	(−0.08**)	(0.08**)	(−0.05**)	(0.05**)
	^+^ETa (W/m^2^)	329.3 ± 2.18	311.4	308.56	309.58	309.26	307.36	309.49	312.32	309.92	311.82
			(−0.09**)	(0.26**)	(−0.12**)	0	(−0.13**)	(−0.01)	–0.01	(0.1**)	(−0.1**)
environmental change from 2000 to 2020	^–^LST Sen’s slope (deg/year)	0.1153 ± 0.0011	0.109	0.109	0.114	0.107	0.113	0.114	0.108	0.112	0.108
			(−0.17**)	(−0.23**)	(0.12**)	(0.08**)	(0.08**)	(0.24**)	(−0.24**)	(0.17**)	(−0.17**)
	^+^ETa Sen’s slope (mm/year)	–0.0063 ± 0.00036	–0.00346	–0.00434	–0.00518	–0.00284	–0.00444	–0.00495	–0.00329	–0.00447	–0.00331
			(−0.07**)	(−0.11**)	(−0.05**)	(0.06**)	(−0.04**)	(0.12**)	(−0.12**)	(0.09**)	(−0.09**)

a WHI: White alone; BLA: Black or African American alone; AIA: American Indian and Alaska native alone; ASI: Asian alone; NHO: native Hawaiian and other Pacific Islander alone; HIS: Hispanic; NHIS: non-Hispanic; NHISW: non-Hispanic White alone; PCOL: People of color. We define racial minorities as groups including Black or African American (BLA), American Indian and Alaska native (AIA), Asian (ASI), native Hawaiian, and Other Pacific Islander (NHO). MHI: median household income; PCI: per capita income; PHBPL: percentage of households below the poverty level; MHPV: median household property values; LST: land surface temperature; ESI: evaporative stress index; ETa: actual evapotranspiration. Note: the correlation of the percentage of races/ethnicities against the variables is in parentheses (*p < 0.05, **p < 0.01). + indicates positive indicators that the higher values are better, and – indicates negative indicators that the lower values are better.

For environmental variables in 2020, White residents experienced the lowest LST among all races, followed by Asians, while other races presented higher heat exposure. Block groups with higher proportions of White and Asian populations showed a significant negative association with LST, indicating cooler surface temperatures. White communities experienced the least severe drought conditions, while other races showed consistently higher drought exposure, as indicated by ESI. The percentage of White and Black or African Americans had a significant positive correlation with ESI, and other races showed negative relationships with the metric, meaning that other races have higher drought situations. ETa, representing consumptive water use, demonstrated a similar distribution pattern to ESI, with the proportion of African Americans showing a positive relationship (*p* < 0.01).

From 2000 to 2020, overall temperatures rose due to global warming and urbanization, reflected in positive LST Sen’s slopes across all races. The fastest LST increases occurred in neighborhoods with higher proportions of American Indian and Alaska Native or Native Hawaiian and Other Pacific Islander populations. White, Black, and Asian communities experienced similar rates of LST increase, with the size of the White and Black communities being negatively associated with LST changes. ETa values decreased over time due to worsening drought conditions, with smaller reductions observed in White and Asian communities. Since plant transpiration, a major component of ETa, generally decreases during drought,^37^ the decrease in ETa implies that the Southwest region experienced worse drought conditions between 2000 and 2020. This interpretation aligns with prior studies, particularly those reporting the severe California droughts from 2014 to 2016.^38–41^

Across contrary groups (Table 1), Hispanics and people of color consistently faced worse social-environmental outcomes than their counterparts. These groups were positively correlated with all negative indicators (*p* < 0.01) and were consistently disadvantaged in comparison, highlighting clear social-environmental inequalities. These findings reveal widespread racial and ethnic disparities in social and environmental conditions. Racial and ethnic minorities are economically vulnerable and disproportionately exposed to heat stress, drought, and water scarcity compared to White and non-Hispanic populations. This study underscores the pressing need to address social-environmental inequalities in the US Southwest.^24,42^

### 3.2 Social-Environmental Differences by Comparing Contrary Groups of Races/Ethnicities

The Wilcoxon test was used to evaluate the hypothesis that intercity means of social-environmental metrics were equal across selected groups (Table 2). The null hypothesis of equal means was rejected (*p* < 0.05) for contrary groups. Positive indicators for Hispanics were significantly lower than those for non-Hispanics, while negative indicators were significantly higher. A similar pattern emerged between racial groups—people of color versus non-Hispanic Whites—with the exception of the ETa Sen’s slope, which showed no significant difference. Comparing differences between contrary groups, Hispanics versus non-Hispanics exhibited larger gaps in median household income and property values but smaller differences in per capita income and percentage of households below the poverty level compared to people of color versus non-Hispanic Whites. The latter group had greater differences in daytime LST and ESI among current environmental indicators.

**Table 2:** Statistical Differences Using Inter-urban Weighted Means Among Races/Ethnicities Across Major US Southwest Cities Using the Wilcoxon Test (Inter-city-46 Urbanized Cities)^a^

		contrary groups	
variables		ethnic groups: Hispanic vs non-Hispanic	racial groups: people of color vs non-Hispanic White
socio-economic conditions in 2020	^+^MHI (K$)	–15.05^**^	–14.14^**^
	^+^PCI (K$)	–9.15^**^	–9.25^**^
	^–^PHBPL	0.039^**^	0.043^**^
	^+^MHPV (K$)	–77.31^**^	–72.38^**^
state of environment in 2020	^–^Daytime LST (deg)	0.617^**^	0.665^**^
	^+^ESI	–0.00966^**^	–0.0131^**^
	^+^ETa (W/m^2^)	–2.83^**^	–1.86^**^
environmental change from 2000 to 2020	^–^LST Sen’s slope (deg/year)	0.00620^**^	0.00366^*^
	^a^ETa Sen’s slope (mm/year)	–0.00167^a^	–0.00116

a MHI: median household income; PCI: per capita income; PHBPL: percentage of household below the poverty level; MHPV: median household property values. LST: land surface temperature; ESI: evaporative stress index; ETa: actual evapotranspiration. The values represent the differences in intercity mean values across the two groups compared. They are calculated by the interurban weighted means of the metrics for Hispanic/People of color subtracted from the metric values of their contrary groups. ^+^indicates positive indicators that the higher values are better, and. ^–^indicates negative indicators that the lower values are better. ^*^ *p* < 0.05. ^**^ *p* < 0.01.

Considering race and ethnicity, social-environmental conditions are consistently worse for minorities and economically disadvantaged groups compared to wealthier populations in the US Southwest. Most indicators showed significant disparities. The Southwest, the hottest and driest US region, faces growing concerns over drought and water scarcity amid climate change.^2,24,43^ ESI and ETa measurements indicate less drought exposure and water scarcity in White, non-Hispanic, and non-Hispanic White neighborhoods. Conversely, minorities and low-income groups face higher drought exposure and water scarcity. Even affluent Asians experience more severe drought and water issues compared to non-Hispanic Whites. This study highlights inequalities in social economy, urban warming, drought, and water use across races/ethnicities, demonstrating the heightened vulnerability of minorities to environmental challenges in the US Southwest. These findings provide compelling evidence of widespread social and environmental inequality in the region. Our weighted mean analyses show that Hispanic and people-of-color groups consistently experienced lower socioeconomic outcomes alongside greater environmental burdens (e.g., higher LST, lower ETa), suggesting that SES disparities likely contribute to modifying environmental inequalities. While this study focuses on racial/ethnic disparities, future work should focus more directly on the analysis of how SES gradients interact with temporal environmental trends to better disentangle the overlapping influences of race, ethnicity, and income.

### 3.3 Environmental Inequalities in Connection with Climate Zones

To better understand environmental inequalities, we examined differences in exposure between contrary groups within distinct climate zones (Arid, Temperate, and Cold, based on Köppen-Geiger classification) (Table 3). For current environmental variables, daytime LST differences between contrary groups were statistically significant (*p* < 0.01), with values exceeding 0.5 °C. The highest differences occurred in Temperate and Cold zones between people of color and non-Hispanic Whites, followed by Arid areas for the same groups. ESI showed significant differences (*p* < 0.01) across all climate zones and contrary groups, while ETa differences varied by climate zone, with significant differences in Arid regions but smaller, nonsignificant differences in Temperate and Cold areas. A similar pattern emerged for LST Sen’s slope, where racial and ethnic minorities showed significantly higher values in Arid neighborhoods compared to their counterparts. However, no significant differences were observed in other climate zones. For ETa Sen’s slope, significant differences were found only between Hispanics and non-Hispanics in Arid climates.

**Table 3:** Statistical Differences in Inter-City Weighted Means among Contrary Groups and Köppen-Geiger Climate Zone Across Major US Southwest Cities Using Wilcoxon Test at City Level (Inter-City-24 Urbanized Cities in Arid, and 22 in Temperate and Cold)^a^

		ethnic groups: Hispanic vs non-Hispanic		racial groups: people of color vs non-Hispanic White	
variables		arid	temperate and cold	arid	temperate and cold
state of environment in 2020	^–^daytime LST (deg)	0.662^**^	0.568^**^	0.632^**^	0.700^**^
	^+^ESI	–0.0106^**^	–0.0106^**^	–0.0117^**^	–0.0144^**^
	^+^ETa (W/m^2^)	–4.83^**^	–0.640	–5.85^**^	2.50
environmental change from 2000 to 2020	^–^LST Sen’s slope from 2000 to 2020 (deg/year)	0.00935^**^	0.00275	0.00635^**^	0.000736
	^+^ETa Sen’s slope from 2000 to 2020 (mm/year)	–0.00237^**^	–0.00091	–0.00101	–0.00121

a LST: land surface temperature; ESI: evaporative stress index; ETa: actual evapotranspiration. ^+^indicates positive indicators that the higher values are better, and ^–^indicates negative indicators that the lower values are better. The values represent the differences in intercity mean values across the two contrary groups. They are calculated by intercity weighted means of the metrics for Hispanic/People of color subtracted from the metric values of their contrary groups within the specific climate zone. ^**^ *p* < 0.01

Environmental inequalities increased over the 21 year study period, as indicated by LST and ETa Sen’s slope differences between races/ethnicities (Table 3). Mean LST and ETa values from 2000 to 2020 (Figure 1) showed clear trends of increasing LSTs in Arid zones across race/ethnicity-dominated neighborhoods, with no notable changes in Temperate and Cold zones. Overall, LST changes mirrored patterns across the study area (Supporting Information S.2), with Hispanic-/people of color-dominated neighborhoods (>75th percentile) consistently exhibiting the highest LSTs, followed by diverse neighborhoods (25th–75th percentile) and non-Hispanic/non-Hispanic White-dominated neighborhoods (<25th percentile). The trends were more pronounced in Arid zones, showing steeper increases and greater differences across racial/ethnic groups. Additional time series for LST and ETa in specific climate zones are provided in the Supporting Information (Figures S8–S11).

**Figure 1:**
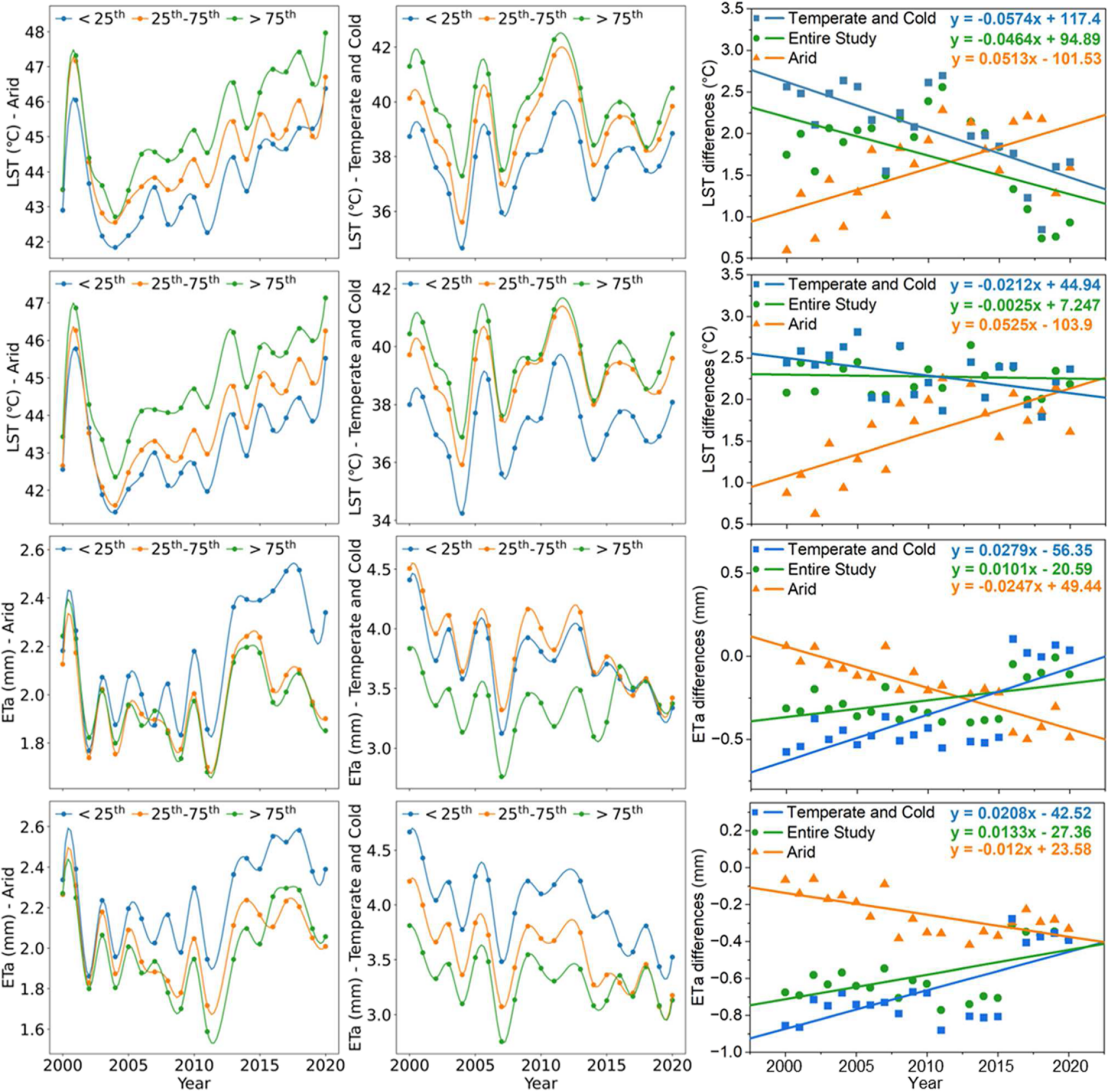
Annual mean LST and ETa values and differences between contrary groups from 2000 to 2020 in arid and temperate and cold regions—(a) LST trends for Hispanic vs non-Hispanic in Arid regions, (b) LST trends for Hispanic vs non-Hispanic in temperate and cold regions, (c) LST differences between Hispanic-dominated and non-Hispanic-dominated neighborhoods, (d) LST trends for people of color vs non-Hispanic White in arid regions, (e) LST trends for people of color vs non-Hispanic White in temperate and cold regions, (f) LST differences between people of color-dominated and non-Hispanic White-dominated neighborhoods, (g) ETa trends for Hispanic vs non-Hispanic in arid regions, (h) ETa trends for Hispanic vs non-Hispanic in temperate and cold regions, (i) ETa differences between Hispanic-dominated and non-Hispanic-dominated neighborhoods, (j) ETa trends for people of color vs non-Hispanic White in arid regions, (k) ETa trends for people of color vs non-Hispanic White in temperate and cold regions, and (l) ETa differences between people of color-dominated and non-Hispanic White-dominated neighborhoods. Note: the <25th, 25th–75th, and >75th percentiles represent neighborhoods with Hispanic/people of color populations lower than the 25th percentile (non-Hispanic-/non-Hispanic White-dominated neighborhoods), between the 25th and 75th percentiles (ethnically/racially diverse neighborhoods), and higher than the 75th percentile (Hispanic-/people of color-dominated neighborhoods). Data for 2012 were omitted due to the unavailability of Landsat images during that time period.

Overall, ETa trends showed more notable declines in Temperate and Cold regions than in Arid areas, differing from the LST trends. However, ETa differences between racial/ethnic groups were more significant in arid regions than in temperate and cold areas. Minority-dominated regions consistently exhibited the lowest ETa values, while non-Hispanic/non-Hispanic White-dominated neighborhoods showed consistently higher ETa values in arid and temperate and cold regions. A similar pattern was observed for people of color versus non-Hispanic Whites in temperate and cold zones. Differences in ETa across racial/ethnic groups were more pronounced in arid zones than in temperate and cold zones. We further quantified changes in environmental inequalities within arid and temperate and cold climate zones (Köppen-Geiger classification). In arid regions, indicators of heat exposure and drought exposure showed significant differences across contrary groups, except for the ETa Sen’s slope between people of color and non-Hispanic Whites (Table 3). In temperate and cold zones, significant differences were detected only for LST, with no significant ETa differences between groups.

We can conclude that in warmer and drier areas, there was greater inequality with respect to exposure to heat stress, drought, and water scarcity issues than in relatively cooler and wetter areas within the US Southwest. In addition, the unexpected outcome was that the ETa of Hispanic-dominated areas was consistently more favorable than non-Hispanic areas in the Temperate and Cold regions from 2000 to 2020, with different results exhibited by areas dominated by people of color versus those dominated by non-Hispanic White.

However, we recognized that regional-scale analyses are subject to the modifiable areal unit problem (MAUP), as aggregating block groups by climate zones can introduce bias due to uneven spatial distributions of sociodemographic and environmental characteristics.^35^ To evaluate the potential impact of MAUP, we compared the distributions of key social-environmental metrics across multiple spatial scales, including the entire study area and individual climate zones. As illustrated in Figures S12–S24, most metrics followed broadly similar distributional patterns across climate zones, thus supporting consistent comparisons. However, certain environmental and biophysical parameters exhibited distinct distributions in specific climate types. For example, while overall LST showed a Gaussian distribution with a slight right skew, the BWK zone displayed a bimodal pattern. Similarly, ESI was generally bimodal but skewed left in Arid, Csa, Csb, BSk, BWh, and BWk zones, and skewed right in Cold and Dfa zones. These can likely be explained by inherent regional differences in climatic and ecological variability rather than inconsistencies in environmental-sociodemographic relationships. These results suggest that the influence of MAUP on our analysis is negligible and support the robustness and reliability of our climate zone-level comparisons and findings. Moreover, to address variation in block group size from aggregating block groups, we employed the complementary method population-weighted means to compare our findings. The population-weighted approach reflects population exposure rather than geographic extent. The consistency between these two analytical strategies strengthens confidence in our interpretation of racial/ethnic disparities across climate zones.

### 3.4 Rising Environmental Inequalities

Trends in environmental inequality changes were assessed using differences in mean LST and ETa Sen’s slopes among racial/ethnic groups. As shown in Figure 1c,f, LST differences were positive, indicating higher LSTs for Hispanic- and people of color-dominated neighborhoods compared to their counterparts over the past 21 years. Overall, LST differences followed the overall trend: temperate and cold regions > entire study area > arid regions. As arid areas had uniformly high temperatures (Figure 1a,b,d,e), warmer regions showed smaller LST differences between contrary groups. However, positive regression coefficients (0.0525 and 0.0513 for racial and ethnic groups, respectively) were significantly higher in Arid regions compared to the entire study area and Temperate and Cold regions (Figure 1c,f), suggesting disparities increased over time in these regions. Specifically, LST differences grew in arid zones but declined in temperate and cold zones, suggesting that disparities in warmer regions intensified while those in cooler regions decreased.

ETa differences were calculated using the same approach as LST differences. Larger negative ETa differences indicate greater disparities. Between 2000 and 2020, ETa values were consistently lower in Arid regions compared to Temperate and Cold regions (Figure 1g,h,j,k). Across all climate zones, disadvantaged groups (Hispanic and people of color) had lower ETa values than their counterparts. ETa disparities declined over time in the entire study area and temperate and cold regions, as indicated by positive linear regression coefficients (Figure 1i,l). In contrast, disparities increased in Arid regions. Notably, disparities between Hispanic- and non-Hispanic-dominated neighborhoods declined more rapidly (coefficient = −0.0247) than those between people of color and non-Hispanic White neighborhoods (coefficient = −0.012) (Figure 1i,l). Arid regions consistently exhibited the largest ETa disparities, suggesting that disadvantaged groups in drier areas face heightened water-related challenges. Additional LST and ETa differences from 2000 to 2020 across contrary groups in specific climate zones are provided in the Supporting Information (Figures S25–S28).

Two reasons might explain the acceleration of inequalities. First, climate change induces more extreme heat in hot regions,^44^ and regions with extreme heat become drier.^24^ This also implies that the impacts of the environment on inequalities are far from independent and static, but become complex with gradual environmental changes. Second, neighborhoods of racial and ethnic minorities are disproportionately affected by land use and land cover changes. For example, green infrastructures either slightly increased or rapidly decreased within racial and ethnic minority neighborhoods during this period.^7,45^ This raises the theory that environmental inequalities increased with increasing income gaps between upper-income, middle-income, and lower-income households during the 21 year period.^46,47^ The evidence of this theory is illustrated by heat and drought stress. This indicates that gradual environmental changes exacerbate existing inequalities between races/ethnicities.

While our study integrates high-resolution satellite products to assess spatial and temporal environmental exposure patterns, the uncertainties are inherent in these data products when used as proxies for complex hydrological processes. For example, ETa is influenced by vegetation density and land surface properties.^29,48^ Similarly, the ESI is a relative measure of drought and may not fully capture soil moisture deficits.^49^ While these indicators offer valuable insights into surface conditions and water dynamics, their application as proxies for human water consumption or long-term drought vulnerability brings uncertainty. Therefore, these metrics should be viewed as indicators of environmental exposure rather than drought conditions.

It is important to consider potential biophysical covariabilities that may influence the observed disparities across climate zones. Factors such as vegetation type, land cover, agricultural intensity, and seasonal water availability can affect LST and ETa.^50,51^ For example, higher ETa values in certain regions may reflect active irrigation or vegetative cover associated with agricultural practices rather than solely population-driven water use.^48^ Similarly, areas with sparse vegetation or arid climates may naturally exhibit lower ETa regardless of community composition.^52^ These environmental and seasonal dependencies may covary with sociodemographic patterns and partially contribute to the spatial variation in environmental exposure. Future research should incorporate detailed vegetation indices, crop type distributions, and land-use and land-cover categories to disentangle these intertwined influences further and refine the attribution of environmental inequality.

## Supplementary Material



## Data Availability

All data sets can be accessed via on Zenodo at http://tinyurl.com/bdsdzj8y, including (a) ECOSTRESS-based LST, ESI, and ETa data sets in 2020, (b) Landsat-based LST and ETa annual mean layers between 2000 and 2020 in the US Southwest, (c) Landsat-based LST and ETa annual Sen’s slope layers, and (d) Block level data including race/ethnicity, social economy, environment (LST, ESI, and ETa), and environmental change (LST and ETa annual Sen’s slope) in 2020 across the US Southwest. A total of 8860 scenes of Landsat data were uploaded (Landsat 4, 5, 7, and 8).

## Notes

Geographic locations of 46 major cities; spatial distributions of land surface temperature (LST), evaporative stress index (ESI), and actual evapotranspiration (ETa) from ECOSTRESS; the spatial distribution of LST and ETa trends from Landsat (2000–2020); flowchart for exploring inequalities; population-based LST and ETa disparities by race/ethnicity and climate zone; socioeconomic and environmental variable distributions across spatial scales; LST and ETa differences by race/ethnicity from 2000 to 2020; list and classification of study cities; data sources and descriptions; outlier exclusion criteria for cities and retention rates by racial/ethnic group; landsat-based LST and ETa retrieval; inequality changes with climate change (PDF)
