# Supplementary material for: Rising Environmental Inequalities and Their Relationship to Racial and Socioeconomic Disparities in the US Southwest
Source: Environ Sci Technol. 2025 Aug 13;59(33):17534–44. doi: 10.1021/acs.est.4c14369 (PMC12393078; doi:10.1021/acs.est.4c14369)
Supplement: Supplementary file 1 [file es4c14369_si_001.pdf]

Supplementary material

Rising environmental inequalities and their relationship to racial and socioeconomic disparities in the US Southwest

*Yuanhui Zhu [1, *], Soe W. Myint [1,2], Danica Schaffer-Smith [3], Daoqin Tong [4], Yuyu Zhou [5,6], Yubin Li [4], Rebecca L. Muenich [7]*

[1]Department of Geography and Environmental Studies, Texas State University, San Marcos, Texas 78666, United States

[2]The Meadows Center for Water and the Environment, San Marcos, Texas 78666, United States

[3]US Fish and Wildlife Service, Raleigh, North Carolina 27636, United States

[4]School of Geographical Sciences and Urban Planning, Arizona State University, Tempe, Arizona 85281, United States

[5]Department of Geography, The University of Hong Kong, Hong Kong 999077, China

[6]Institute for Climate and Carbon Neutrality, The University of Hong Kong, Hong Kong 999077, China

[7]Department of Biological and Agricultural Engineering, University of Arkansas, Fayetteville, Arkansas 72701, United States

Corresponding author: Yuanhui Zhu (jta90@txstate.edu)

Contents Summary:

- Total pages: 38
- Figures: 29 (Figures S1–S29)
- Tables: 4 (Tables S1–S4)

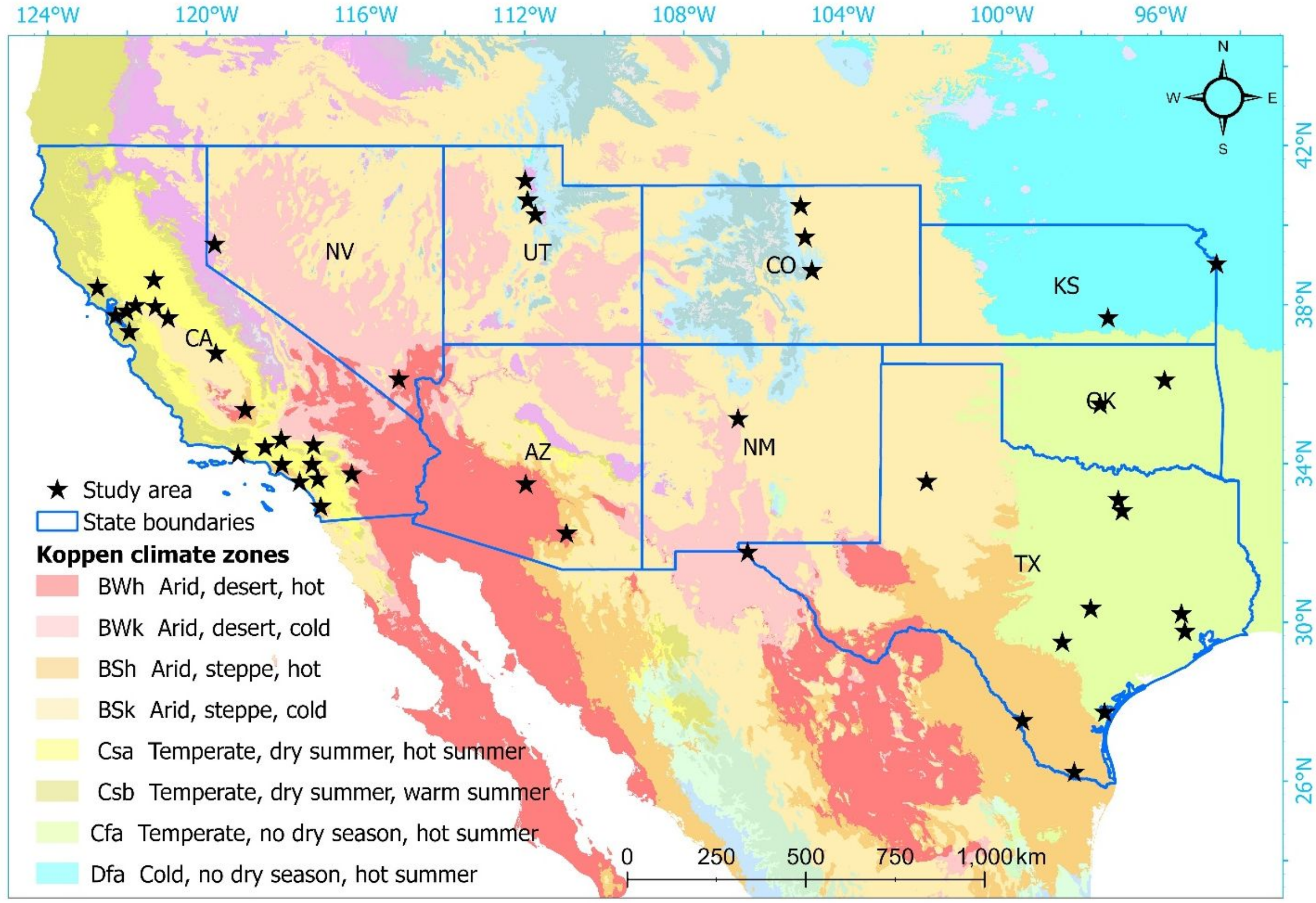

Figure S1 Geographic locations of 46 major cities in the U.S. Southwest overlaid with Köppen-Geiger climate zone classifications for the study areas.

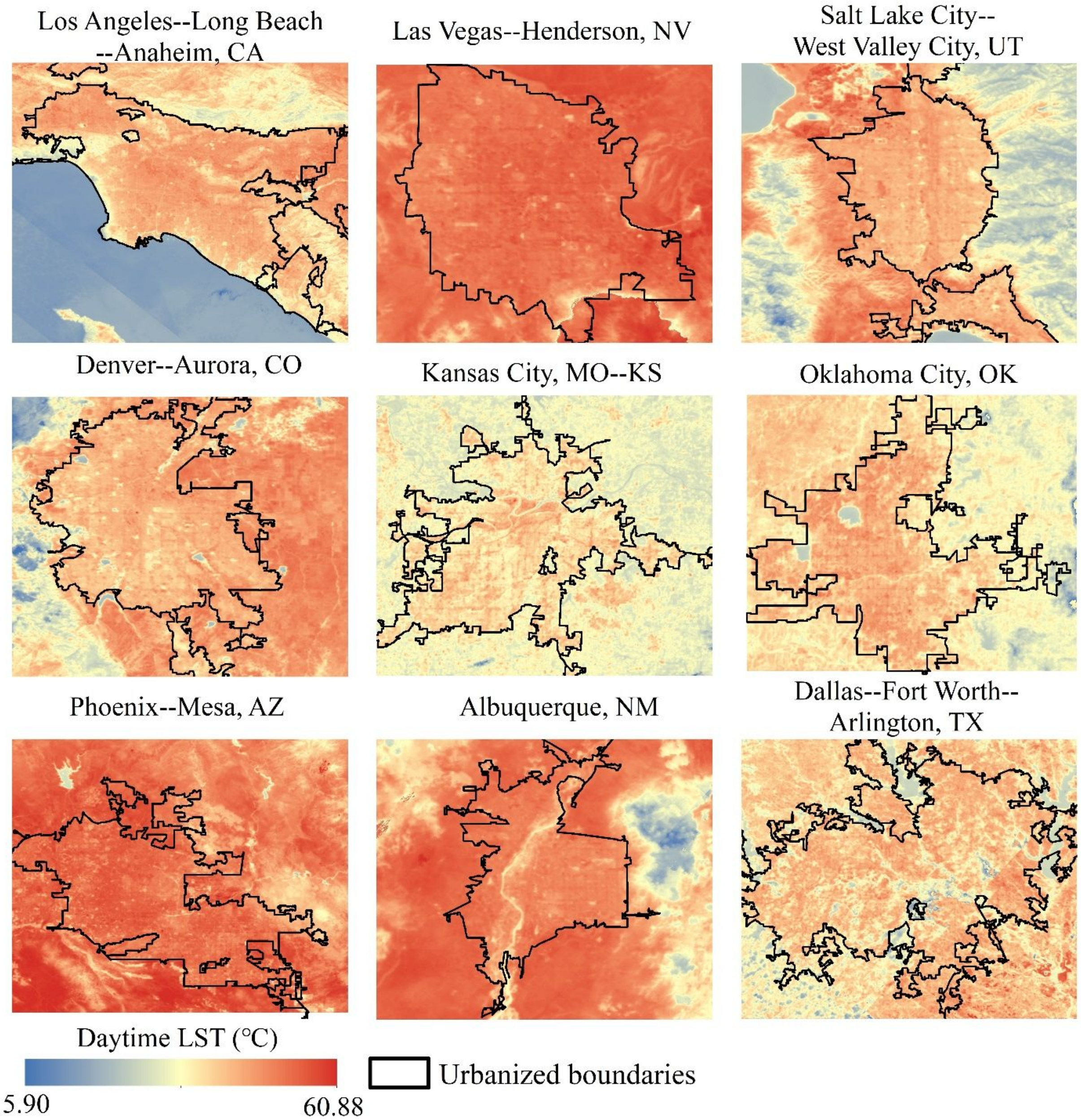

Figure S2 The spatial distribution of mean daytime land surface temperatures (LSTs) derived from ECOSTRESS data in 2020 over the representative regions within our study areas. All the datasets employed in this study are available for public use on Zenedo at http://tinyurl.com/bdsdzj8y.

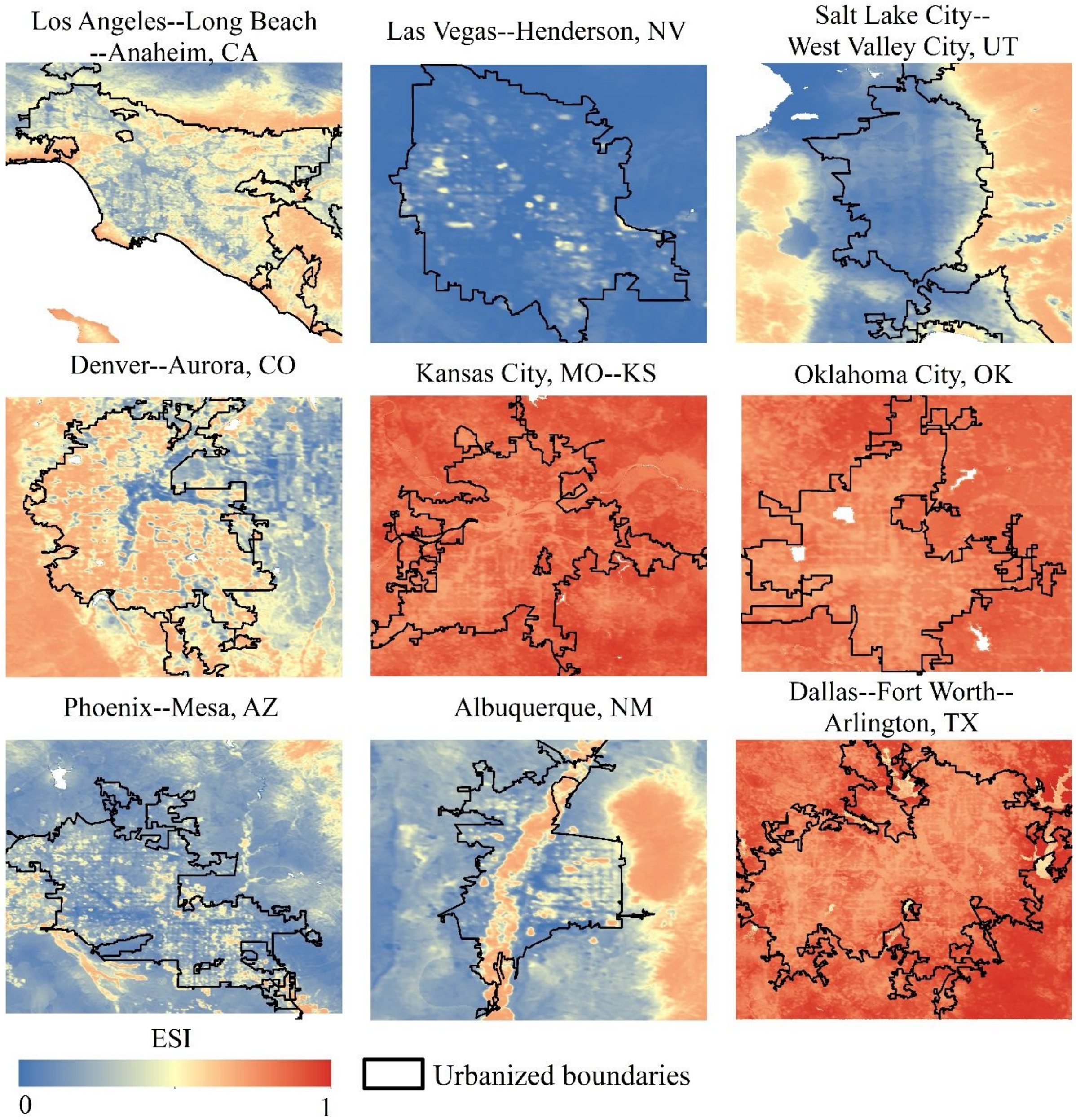

Figure S3 The spatial distribution of mean evaporative stress index (ESI) derived from ECOSTRESS data in 2020 over the representative regions within our study areas. All the datasets employed in this study are available for public use on Zenodo at http://tinyurl.com/bdsdzj8y.

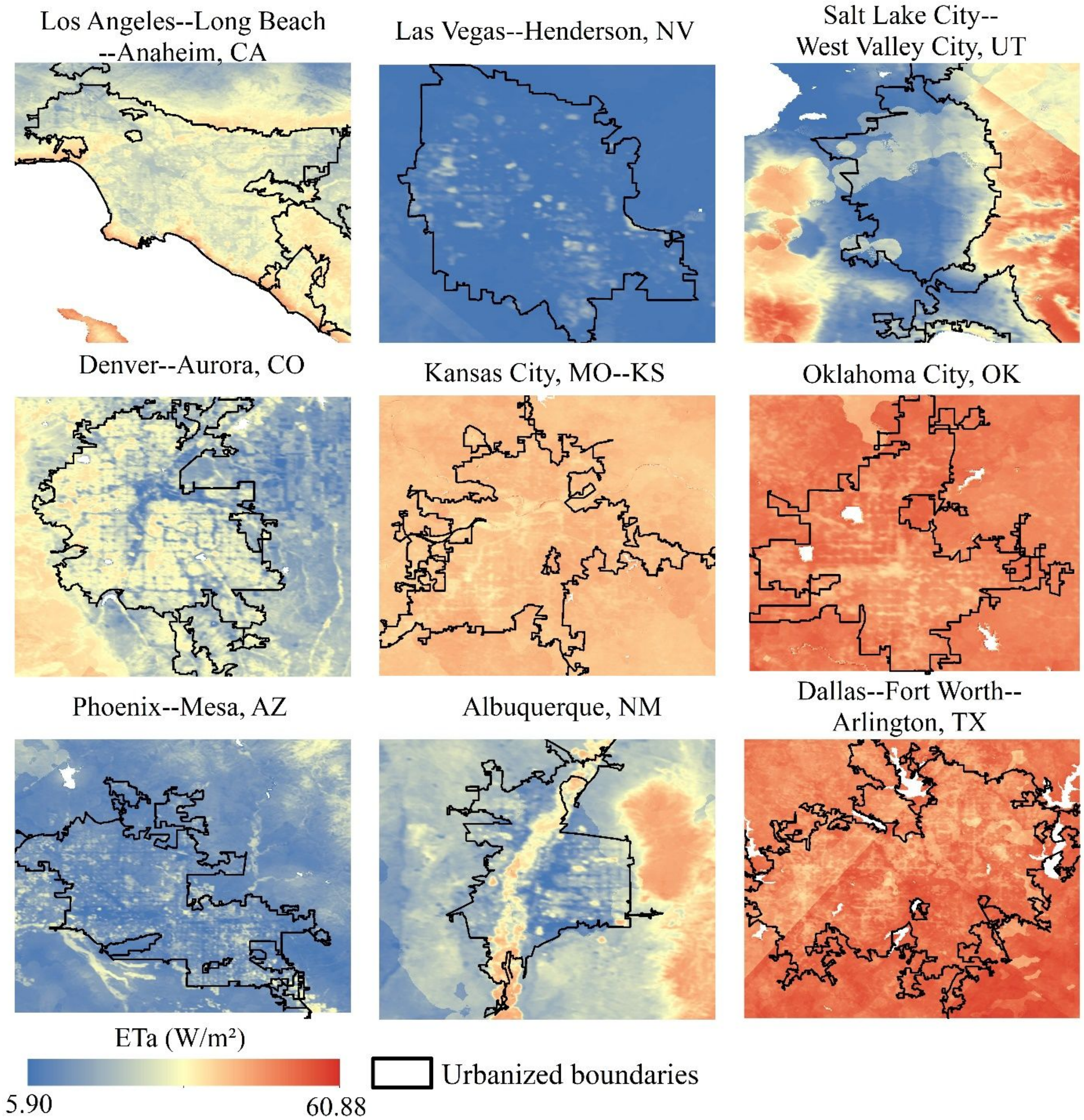

Figure S4 The spatial distribution of mean actual evapotranspiration (ETa) derived from ECOSTRESS data in 2020 over the representative regions within our study areas. All the datasets employed in this study are available for public use on Zenedo at http://tinyurl.com/bdsdzj8y.

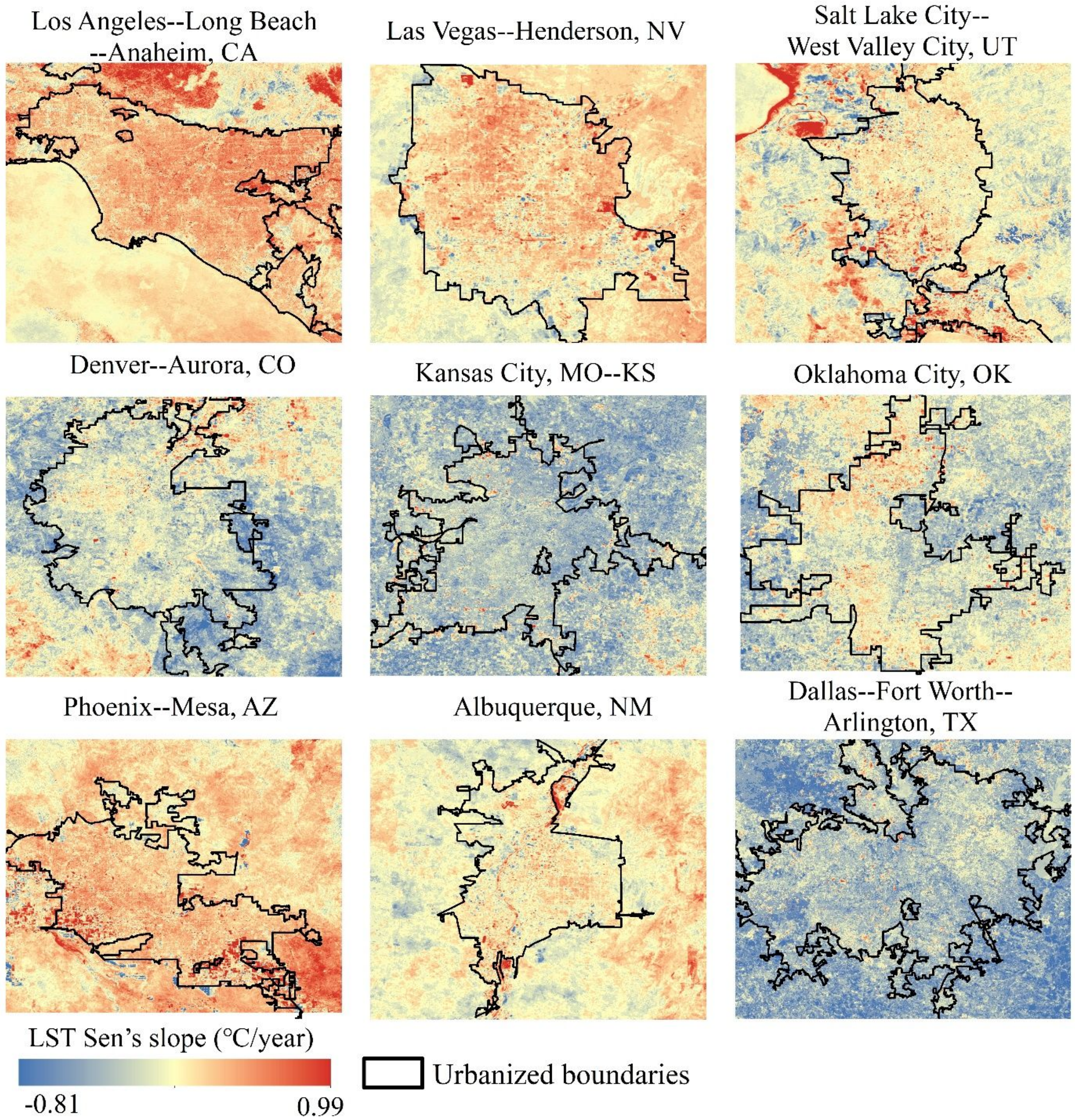

Figure S5 The spatial distribution of Sen's slope values based on annual land surface temperatures (LSTs) layers derived from Landsat images between 2000 and 2020 over the representative regions within our study areas. All the datasets employed in this study are available for public use on Zenedo at http://tinyurl.com/bdsdzj8y.

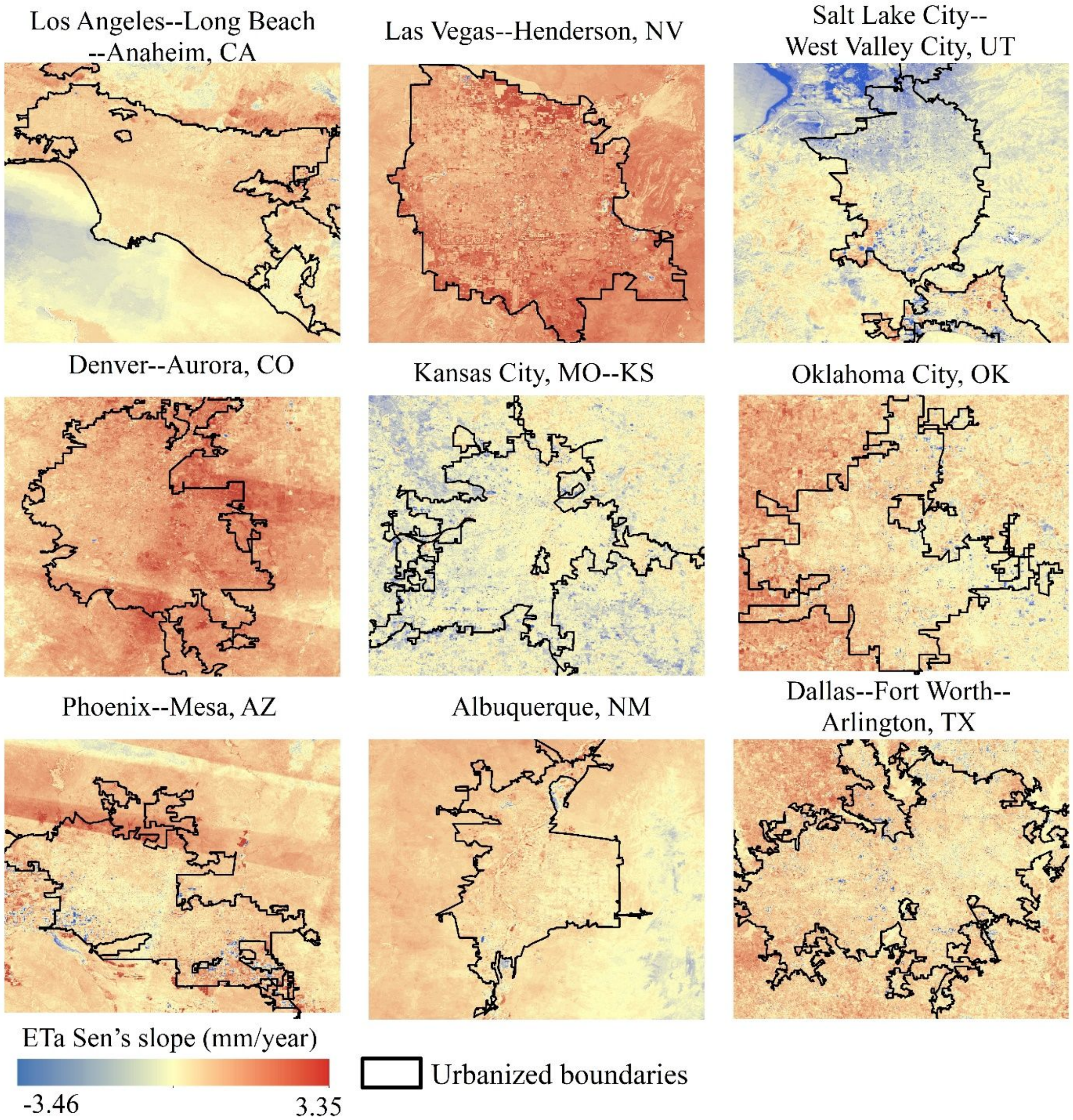

Figure S6 The spatial distribution of Sen's slope values based on annual actual evapotranspiration (ETa) layers derived from Landsat images between 2000 and 2020 over the representative regions within our study areas. All the datasets employed in this study are available for public use on Zenedo at http://tinyurl.com/bdsdzj8y.

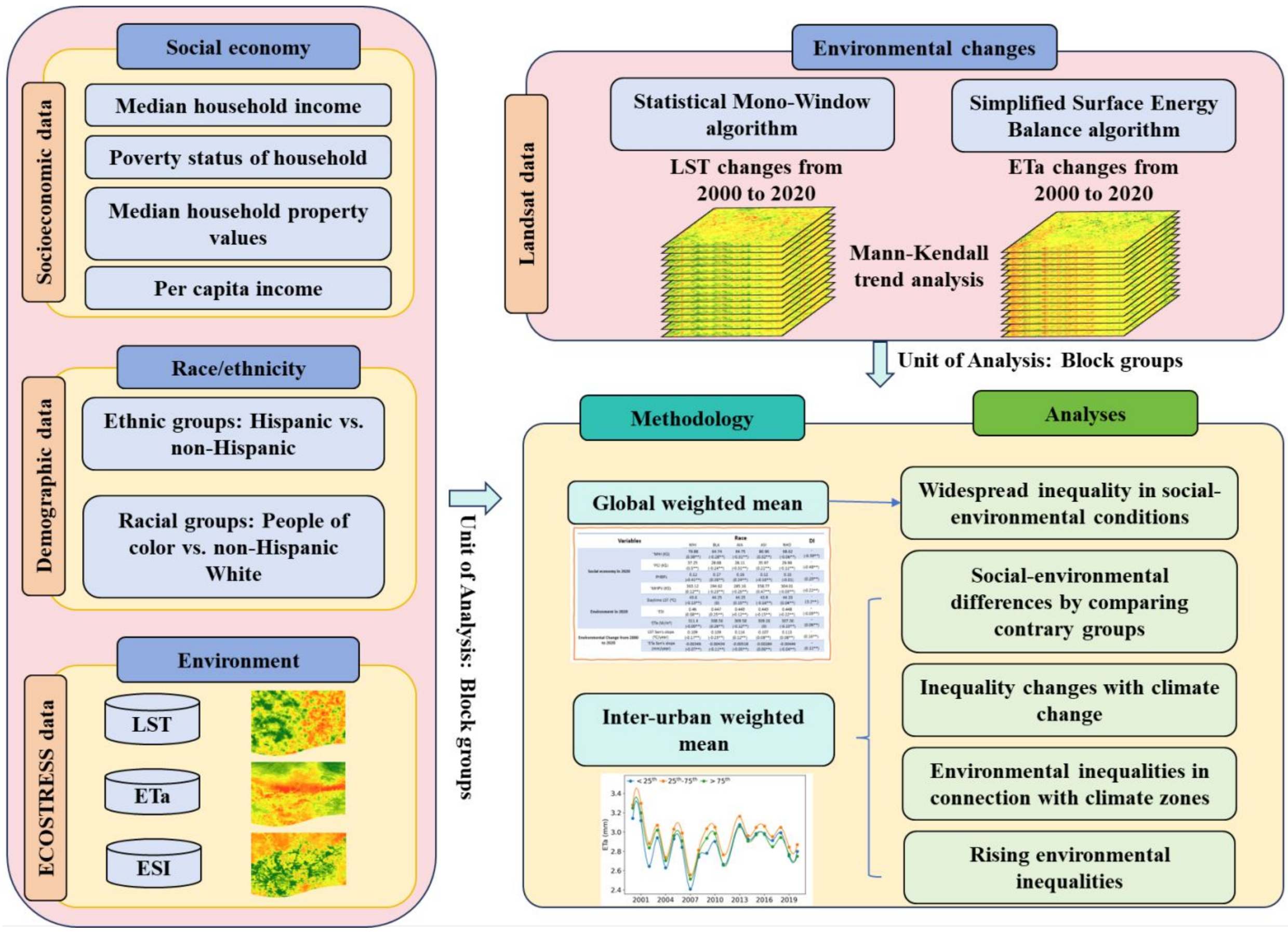

Figure S7 Flowchart for exploring inequalities between races/ethnicities in the US Southwest

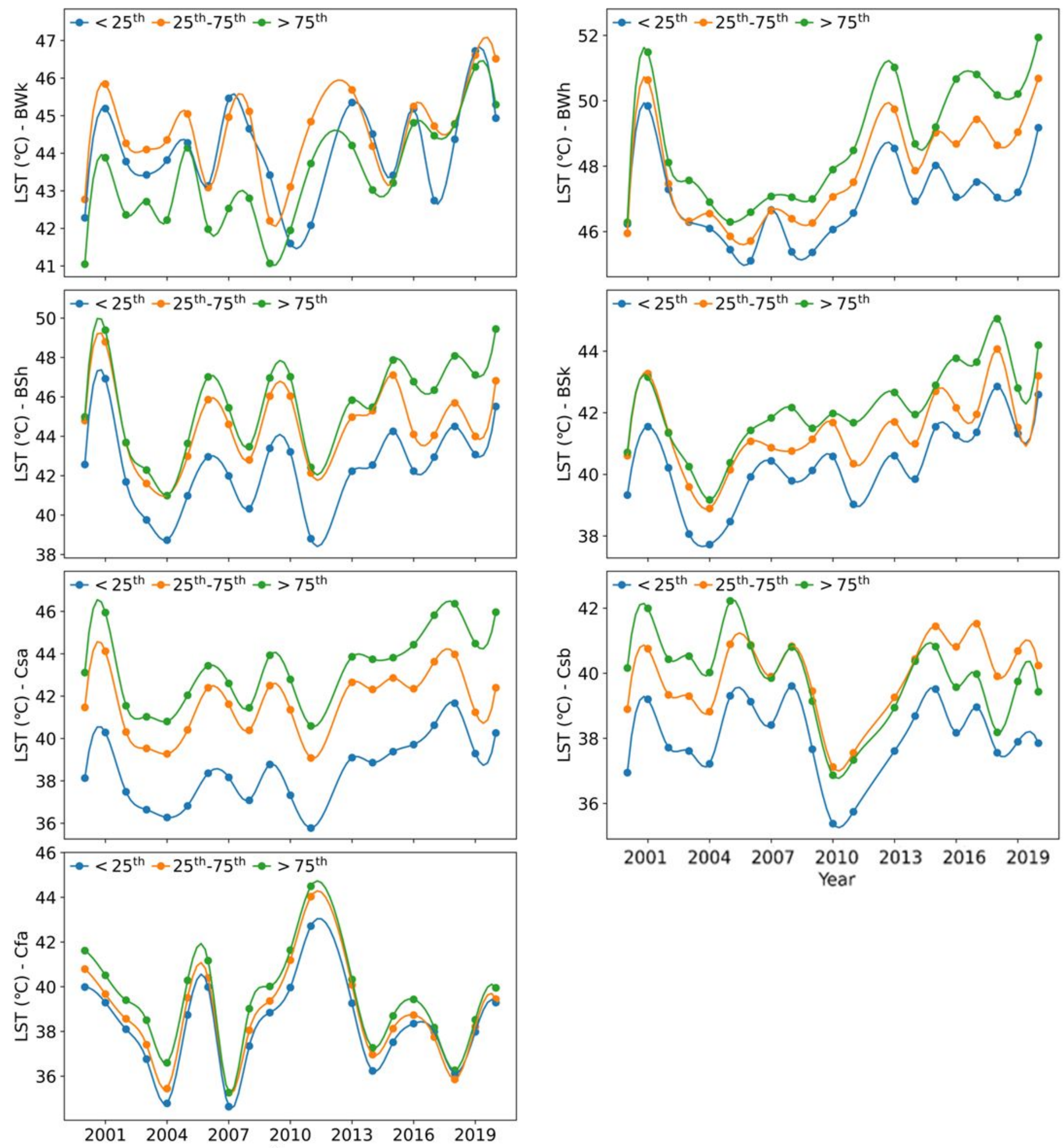

Figure S8 The time series of LST annual means from 2000 to 2020 in connection with specific climate zones across different areas for Hispanic vs. non-Hispanic. Note: <25th, 25th—75th, and >75th percentiles represent the areas with Hispanic population lower than 25th (non-Hispanic-dominated neighborhoods), between 25th and 75th (ethnically diverse neighborhoods), and higher than 75th percentiles (Hispanic-dominated neighborhoods) at the city level, respectively. Climate zones: BWk-Arid, desert, hot; BWh—Arid, desert, cold; BSh—Arid, steppe, hot; BSk—Arid, steppe, cold; Csa—Temperate, dry summer, hot summer; Csb—Temperate, dry summer, warm summer; Cfa—Temperate, no dry season, hot summer; Dfa—Cold, no dry season, hot summer.

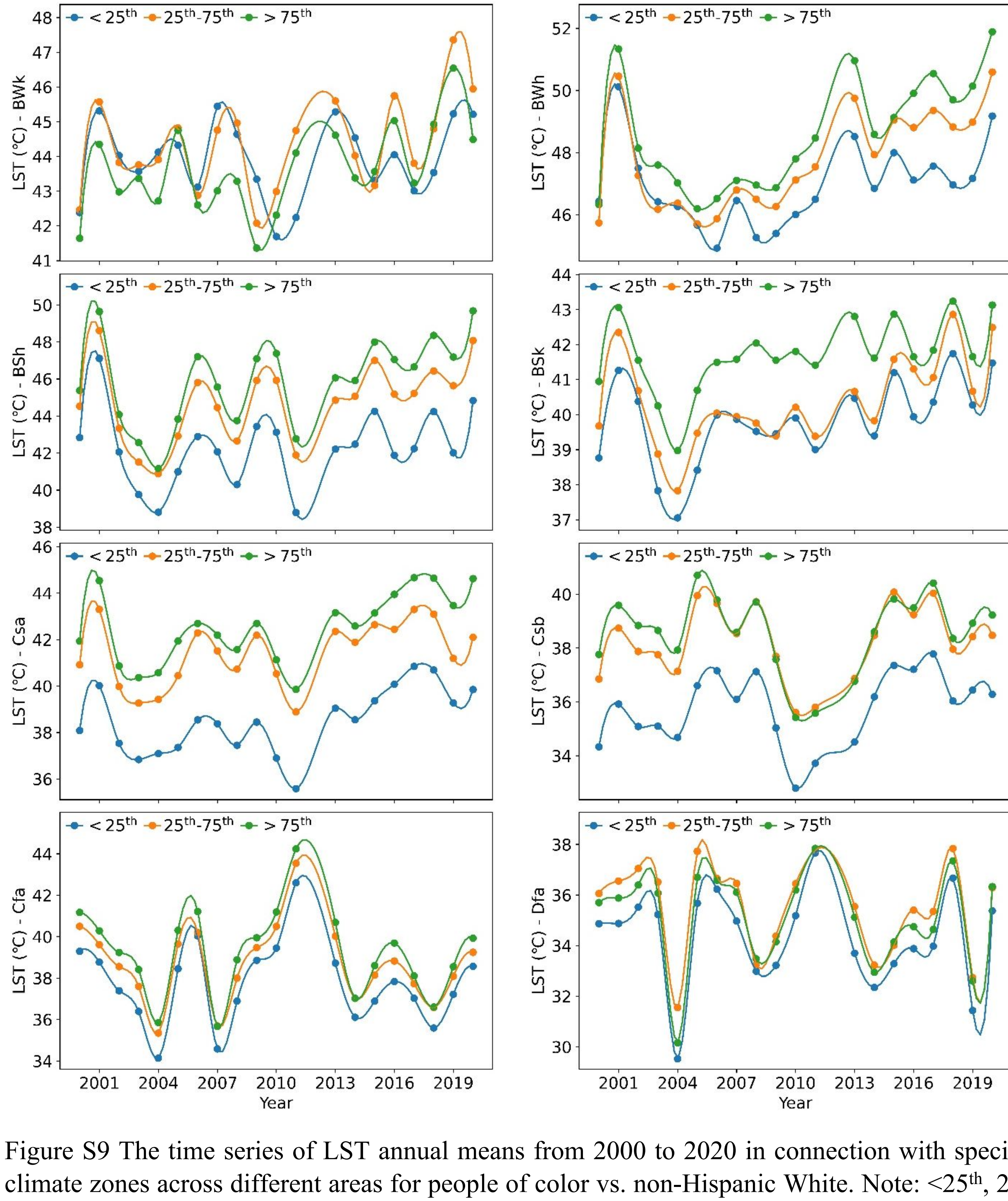

Figure S9 The time series of LST annual means from 2000 to 2020 in connection with specific climate zones across different areas for people of color vs. non-Hispanic White. Note: <25th, 25th – 75th, and >75th percentiles represent the areas with a population of Hispanic lower than 25th (non-Hispanic White-dominated neighborhoods), between 25th and 75th (racially diverse neighborhoods) and higher than 75th percentiles (people of color-dominated neighborhoods) at the city level, respectively. Climate zones: BWk-Arid, desert, hot; BWh—Arid, desert, cold; BSh—Arid, steppe, hot; BSk—Arid, steppe, cold; Csa—Temperate, dry summer, hot summer; Csb—Temperate, dry

summer, warm summer; Cfa—Temperate, no dry season, hot summer; Dfa—Cold, no dry season, hot summer.

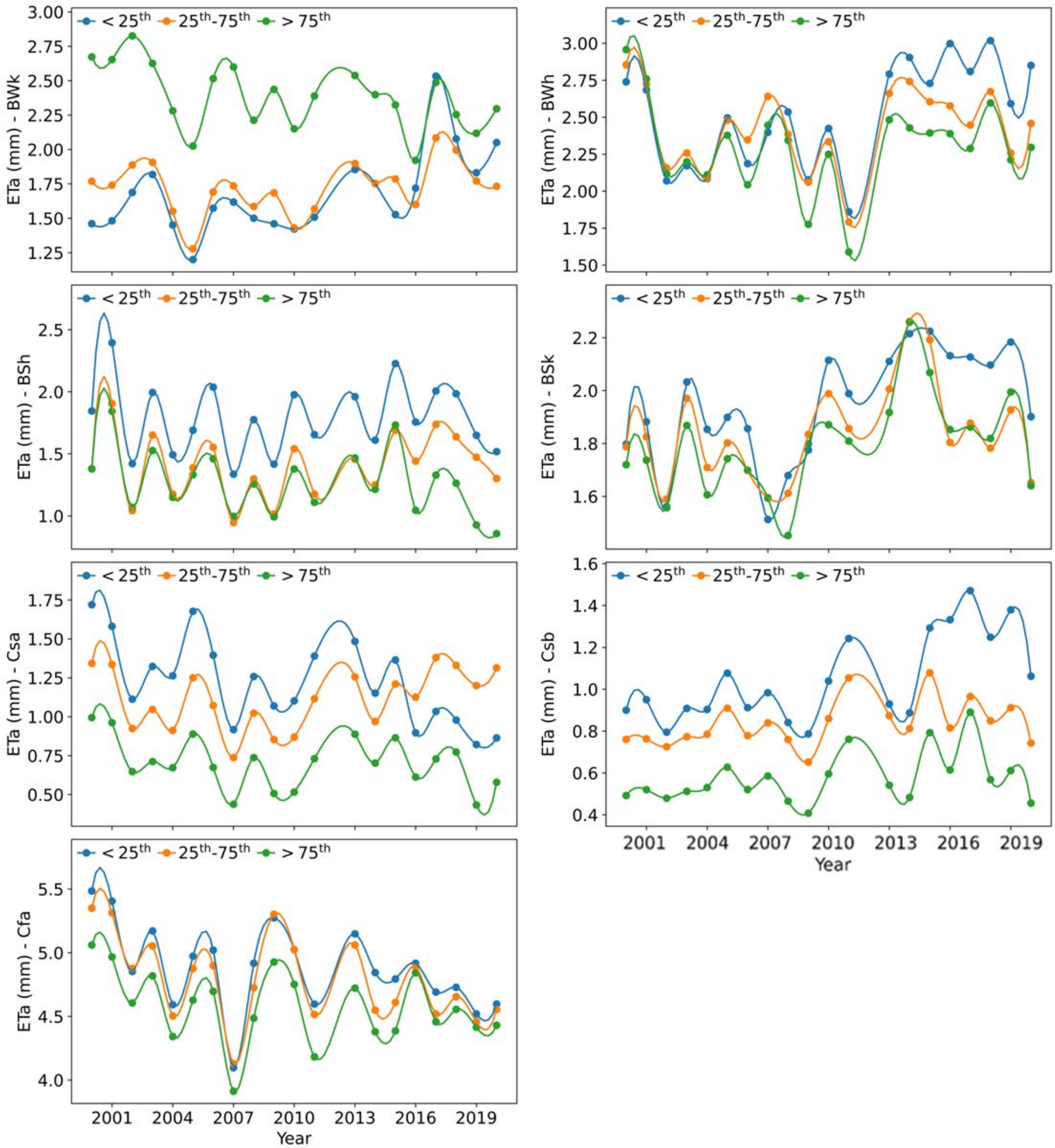

Figure S10 The time series of ETa annual means from 2000 to 2020 in connection with specific climate zones across different neighborhoods for Hispanic vs. non-Hispanic. Note: <25th, 25th – 75th, and >75th percentiles represent the areas with Hispanic population lower than 25th (non-Hispanic-dominated neighborhoods), between 25th and 75th (ethnically diverse neighborhoods), and higher than 75th percentiles (Hispanic-dominated neighborhoods) at the city level, respectively. Climate zones: BWk-Arid, desert, hot; BWh—Arid, desert, cold; BSh—Arid, steppe, hot; BSk—

Arid, steppe, cold; Csa—Temperate, dry summer, hot summer; Csb—Temperate, dry summer, warm summer; Cfa—Temperate, no dry season, hot summer; Dfa—Cold, no dry season, hot summer.

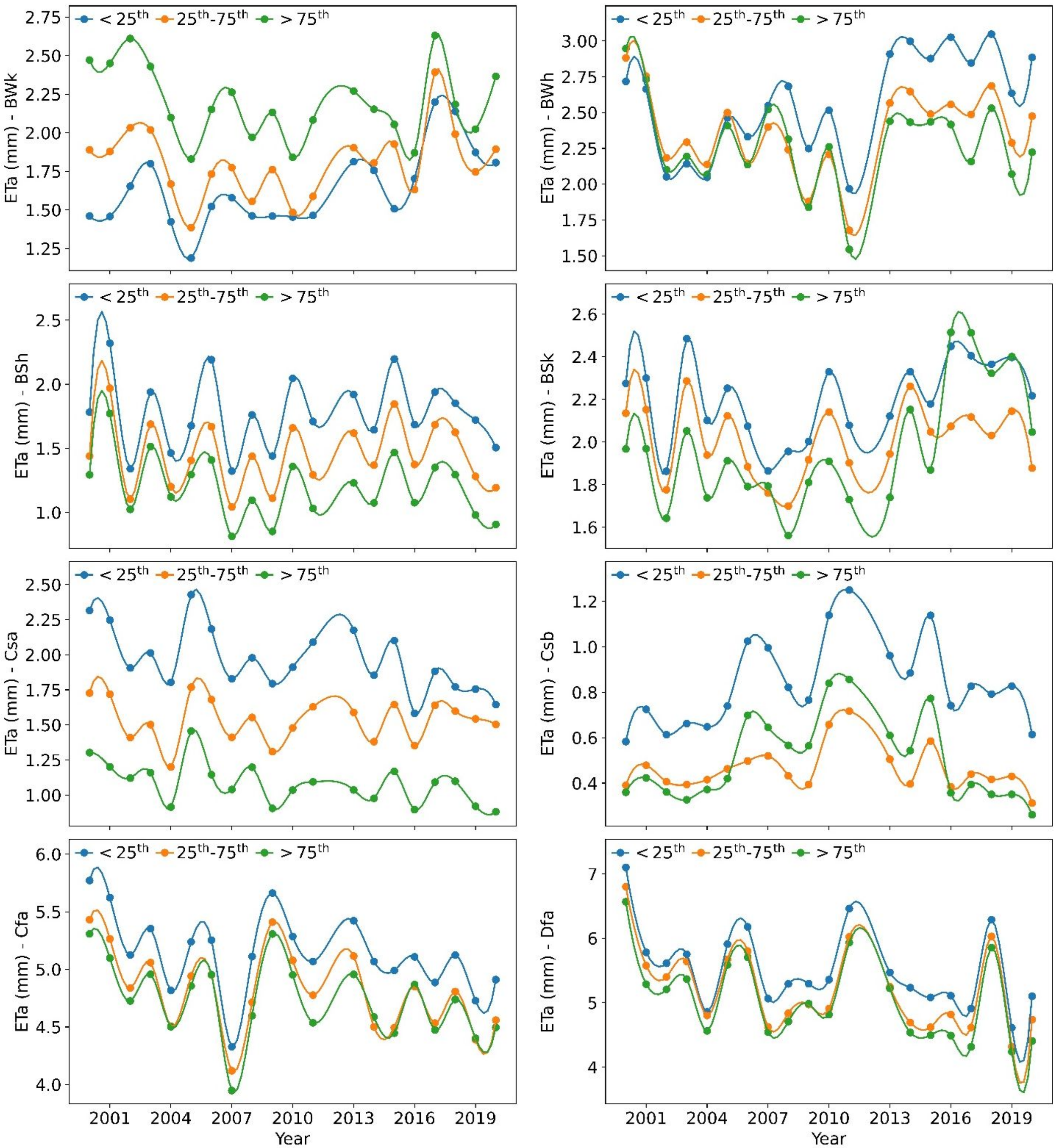

Figure S11 The time series of ETa annual means from 2000 to 2020 in connection with specific climate zones across different neighborhoods for people of color vs. non-Hispanic White. Note: $<25^{th}$, 25th – 75th, and $>75^{th}$ percentiles represent the areas with a population of Hispanic lower than 25th (non-Hispanic White-dominated neighborhoods), between 25th and 75th (racially diverse

neighborhoods) and higher than 75th percentiles (people of color-dominated neighborhoods) at the city level, respectively. Climate zones: BWk-Arid, desert, hot; BWh—Arid, desert, cold; BSh—Arid, steppe, hot; BSk—Arid, steppe, cold; Csa—Temperate, dry summer, hot summer; Csb—Temperate, dry summer, warm summer; Cfa—Temperate, no dry season, hot summer; Dfa—Cold, no dry season, hot summer.

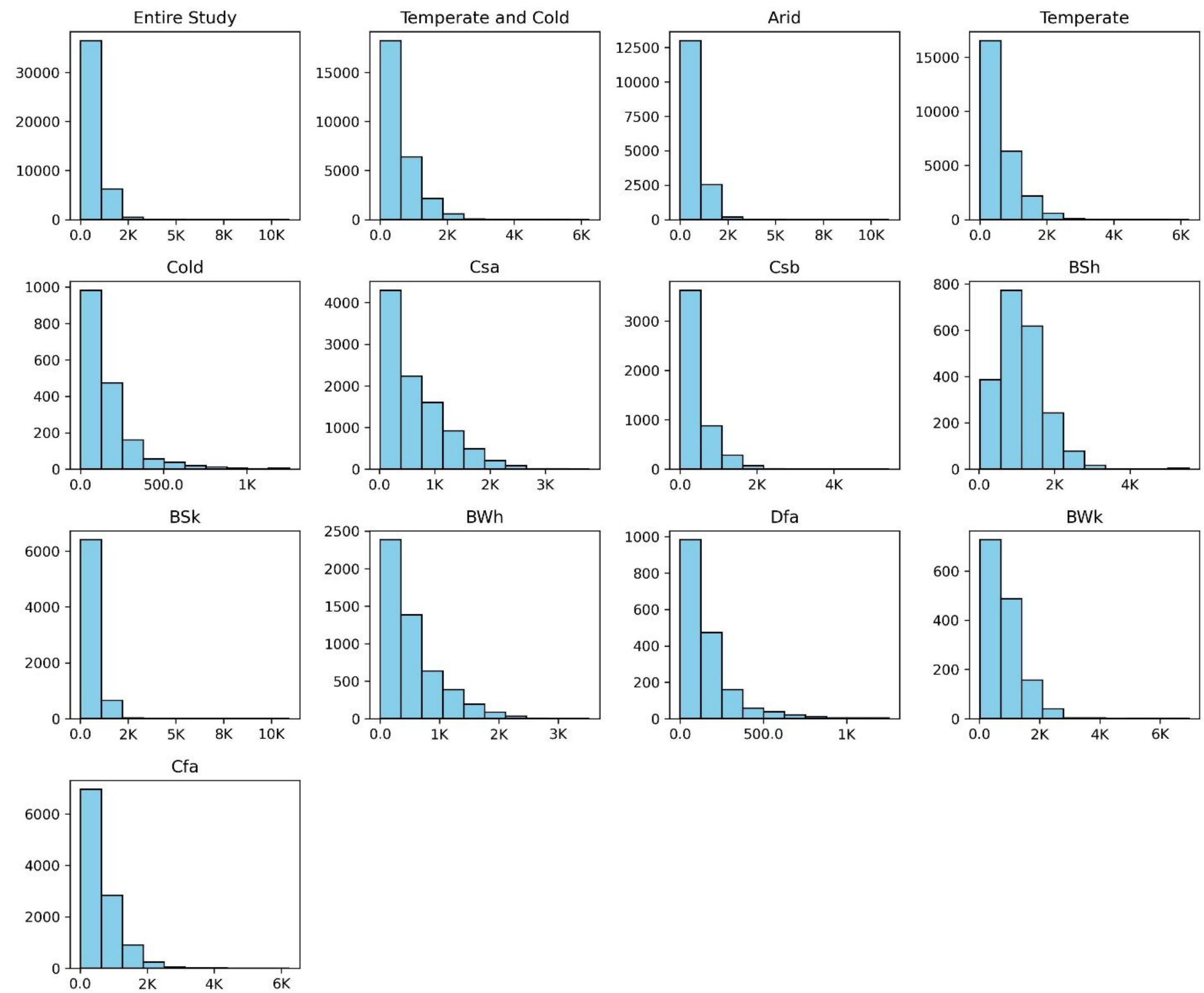

Figure S12 The distribution of the Hispanic population across spatial scales (entire study area and different climate zones).

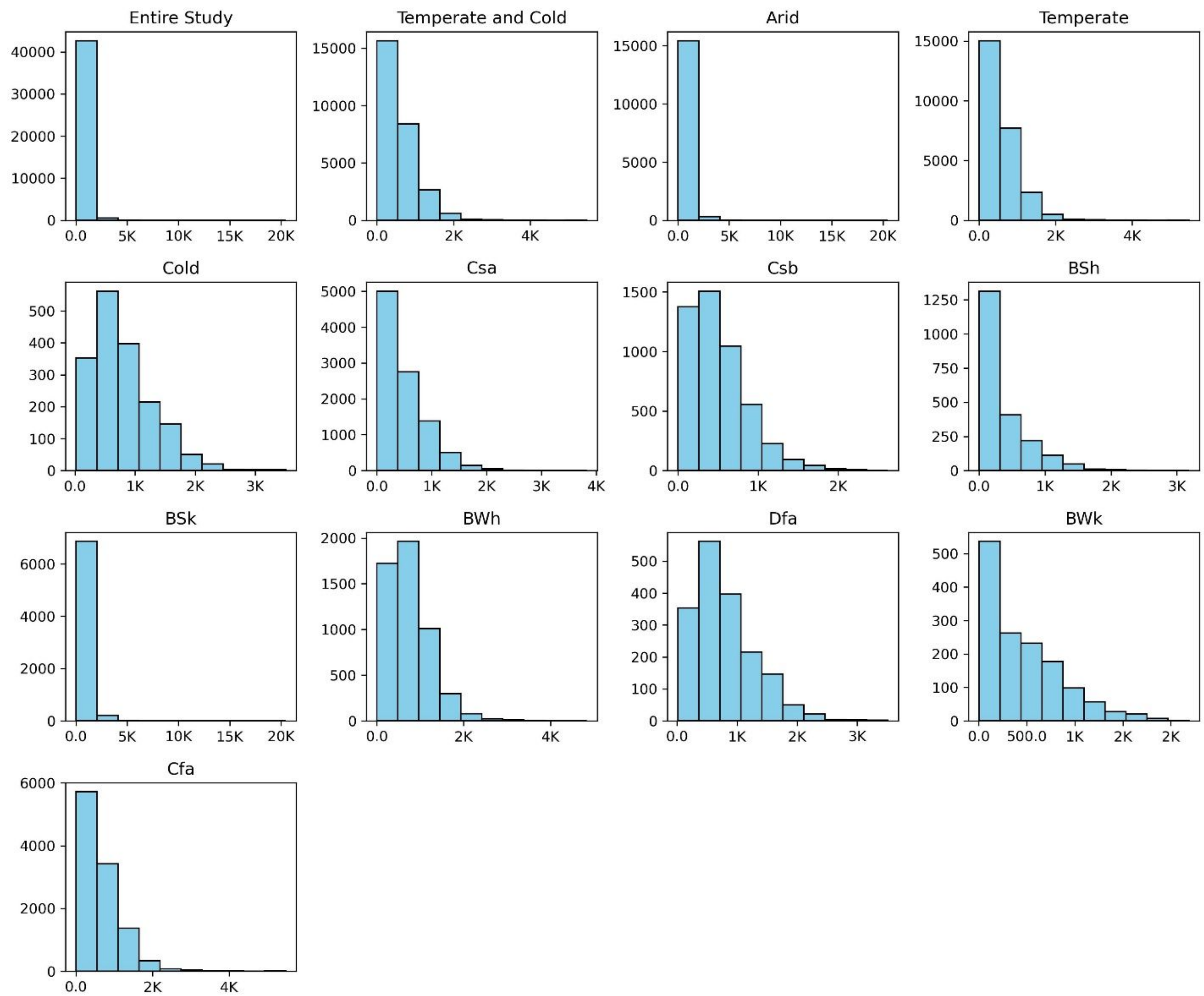

Figure S13 The distribution of the non-Hispanic population across spatial scales (entire study area and different climate zones).

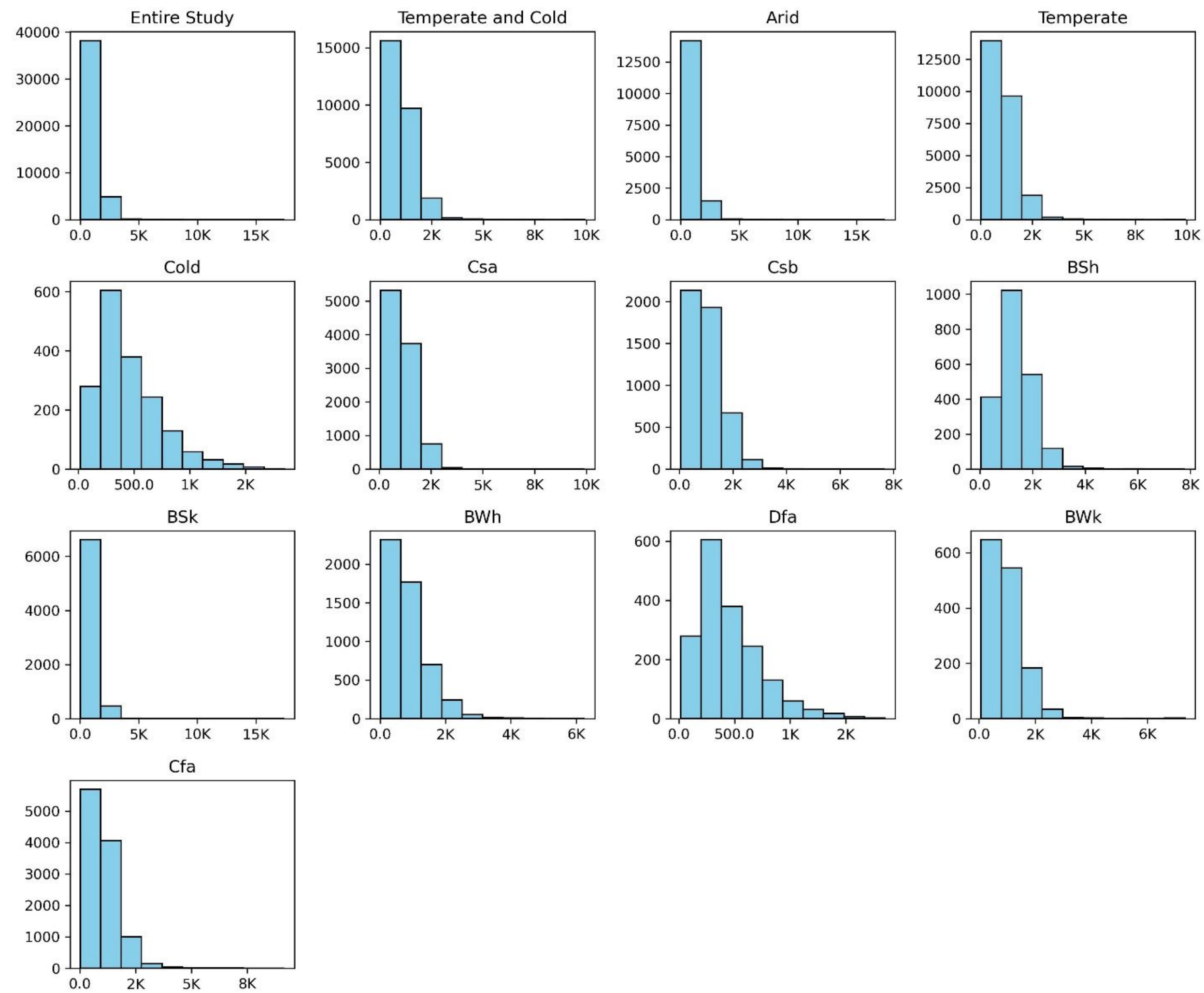

Figure S14 The distribution of the people of color population across spatial scales (entire study area and different climate zones).

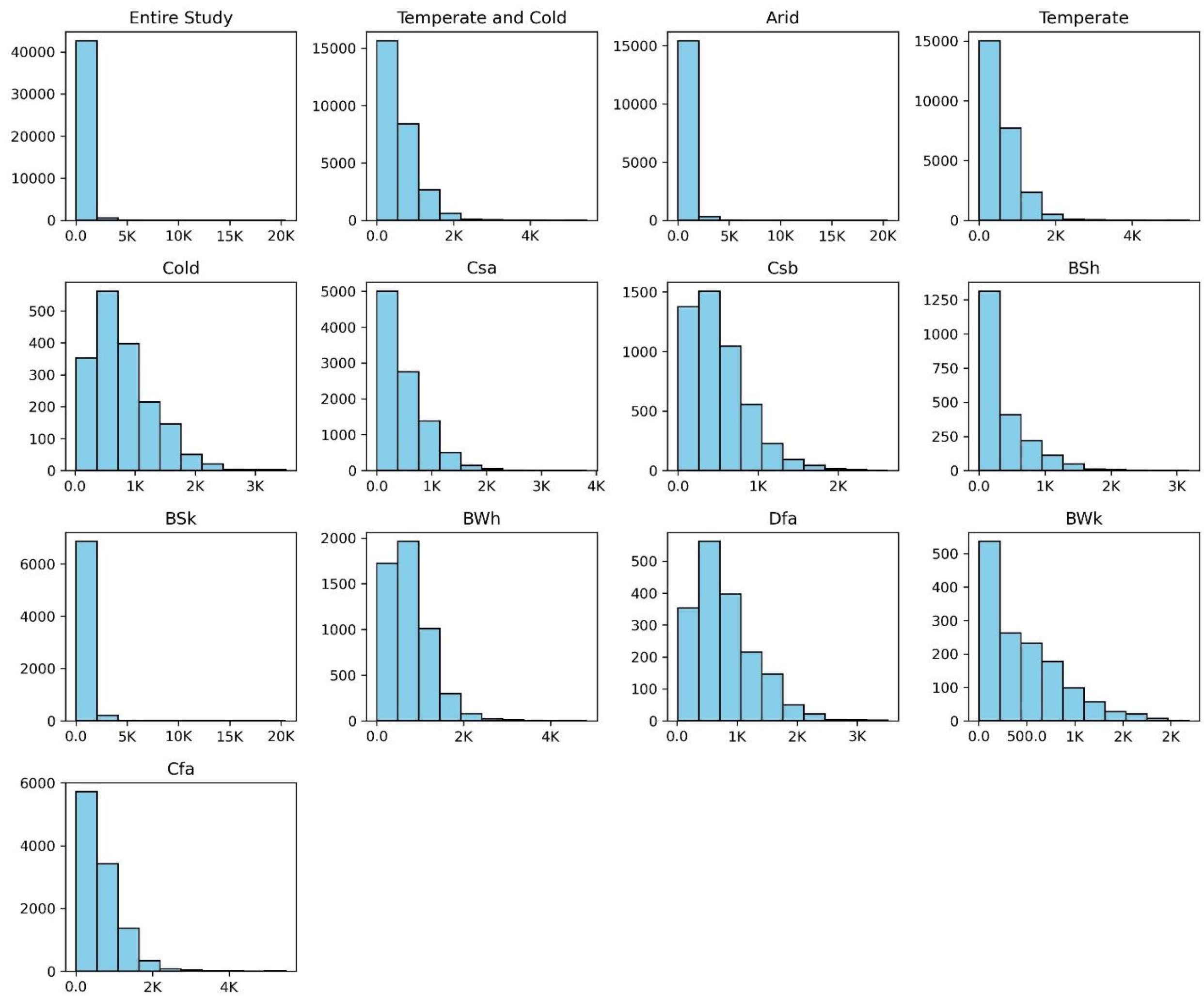

Figure S15 The distribution of the non-Hispanic White population across spatial scales (entire study area and different climate zones).

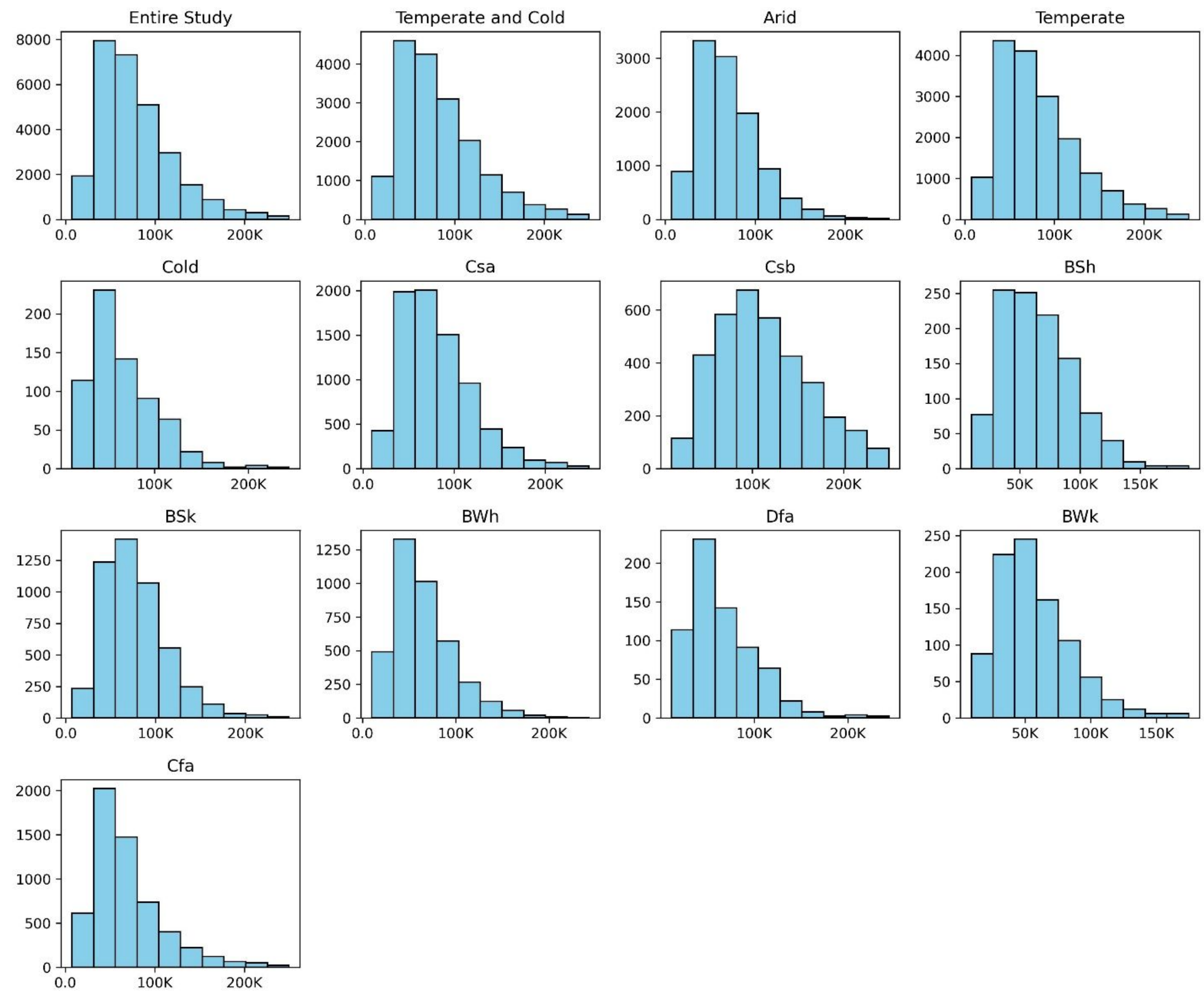

Figure S16 The distribution of the median household income across spatial scales (entire study area and different climate zones).

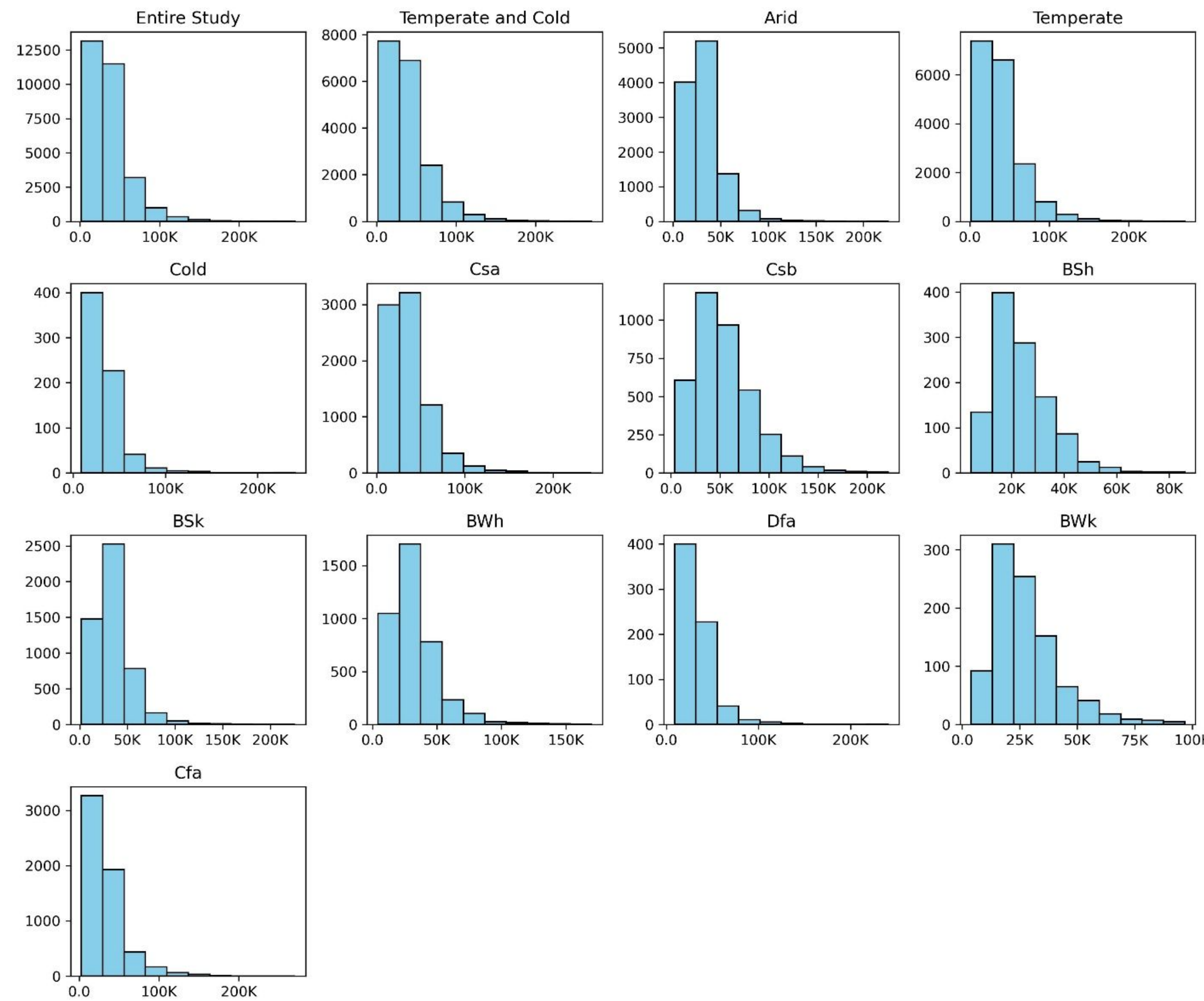

Figure S17 The distribution of per capita income across spatial scales (entire study area and different climate zones).

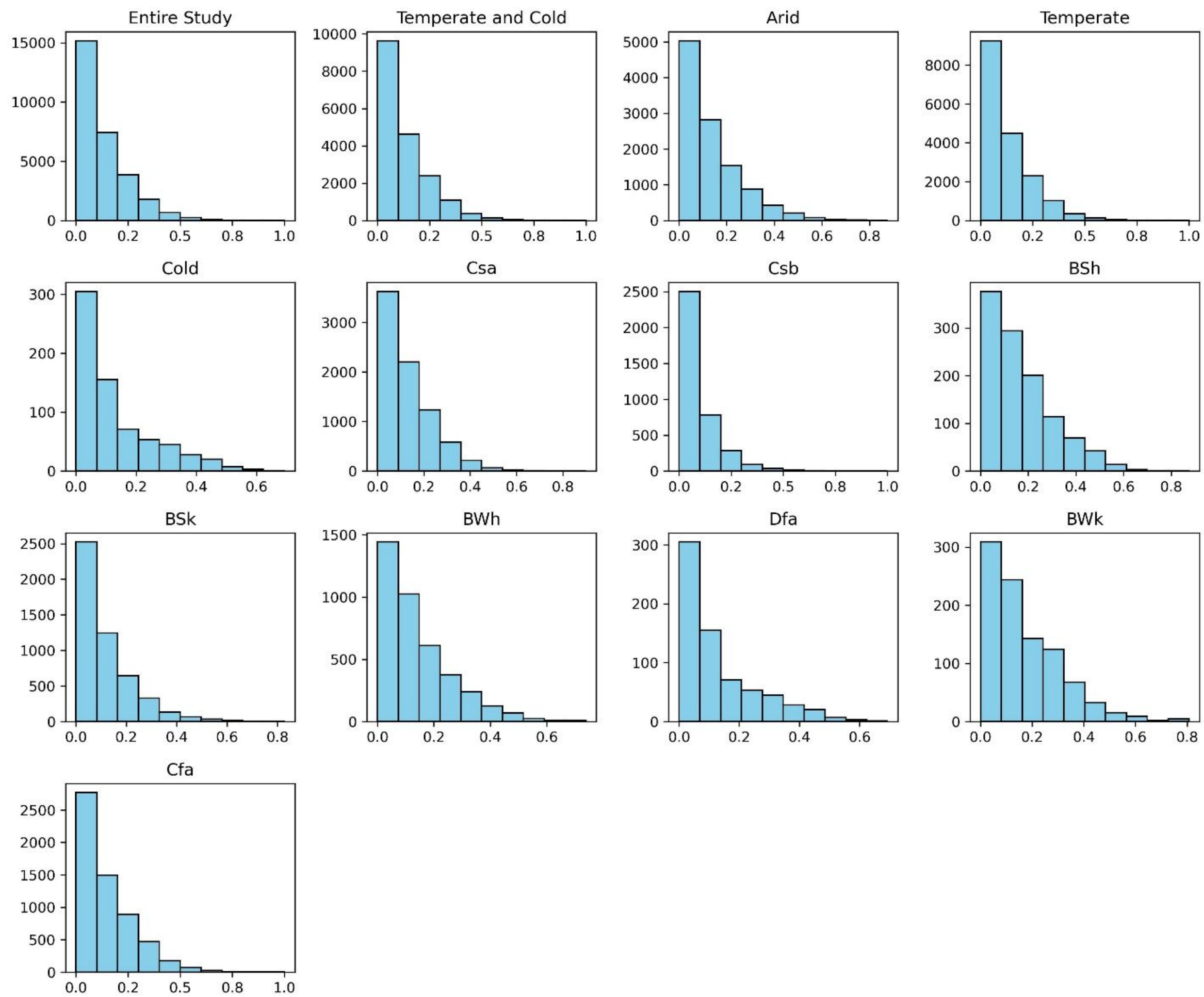

Figure S18 The distribution of the percentage of household below the poverty level across spatial scales (entire study area and different climate zones).

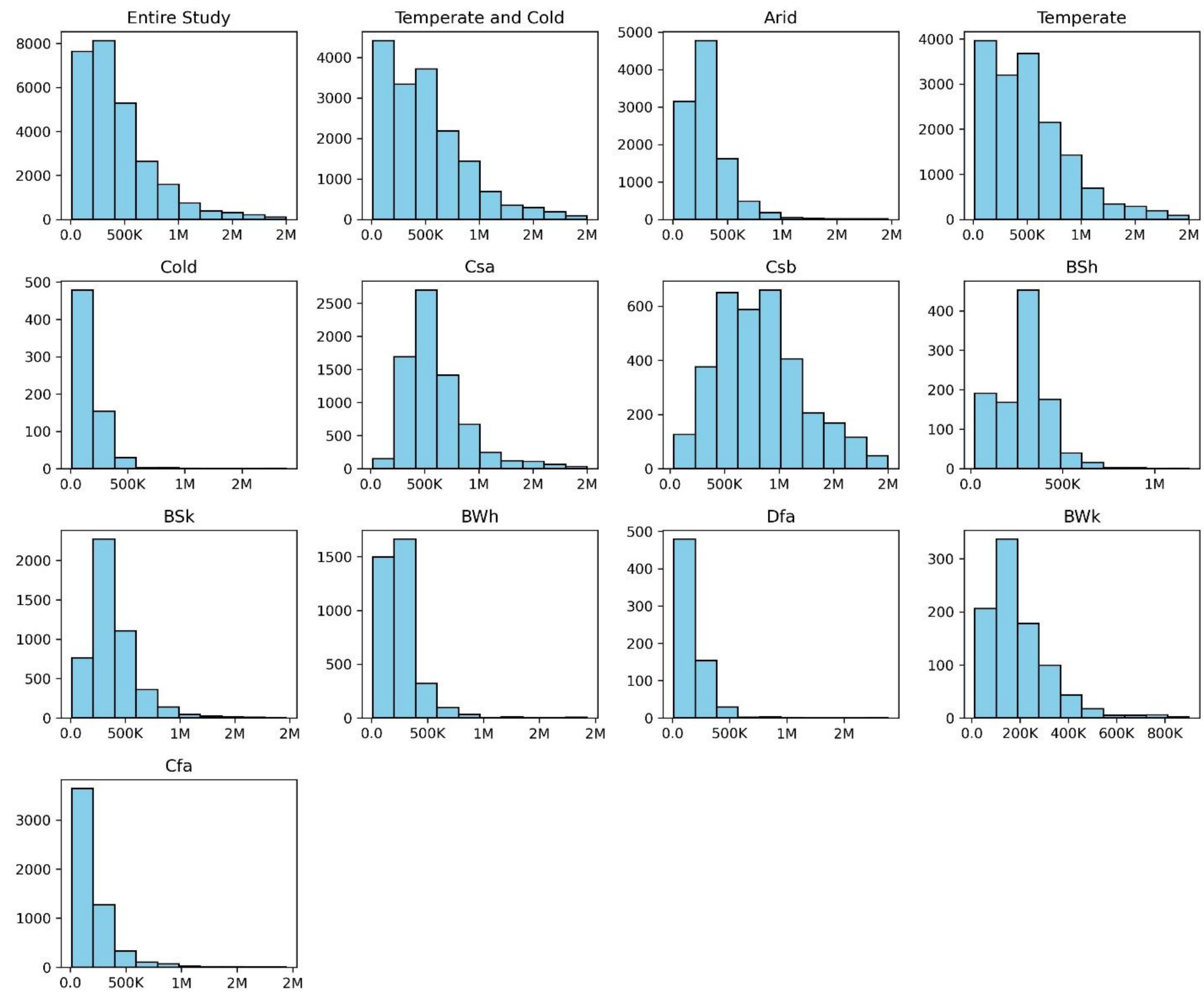

Figure S19 The distribution of the median household property values across spatial scales (entire study area and different climate zones).

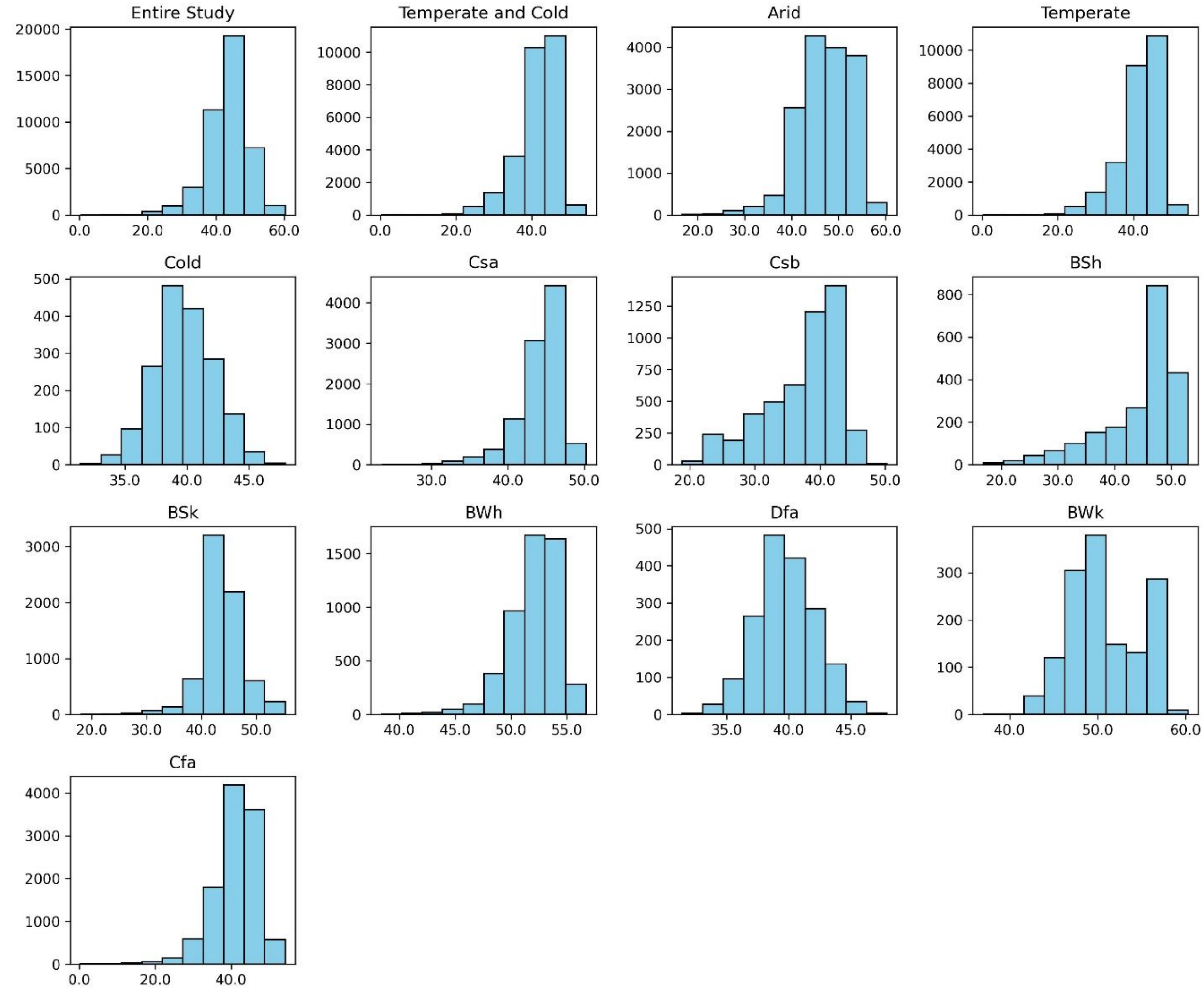

Figure S20 The distribution of land surface temperature (℃) across spatial scales (entire study area and different climate zones).

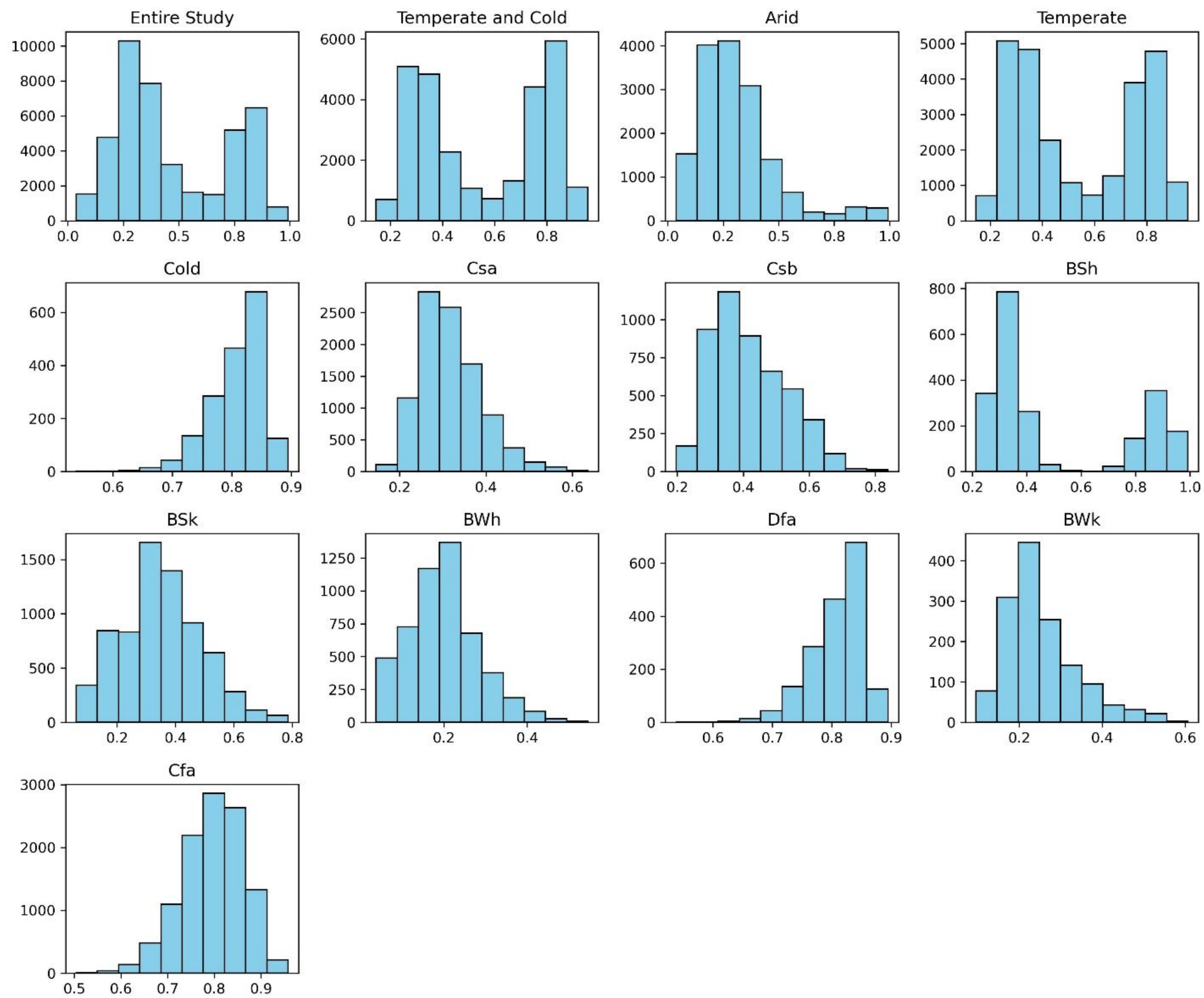

Figure S21 The distribution of evaporative stress index across spatial scales (entire study area and different climate zones).

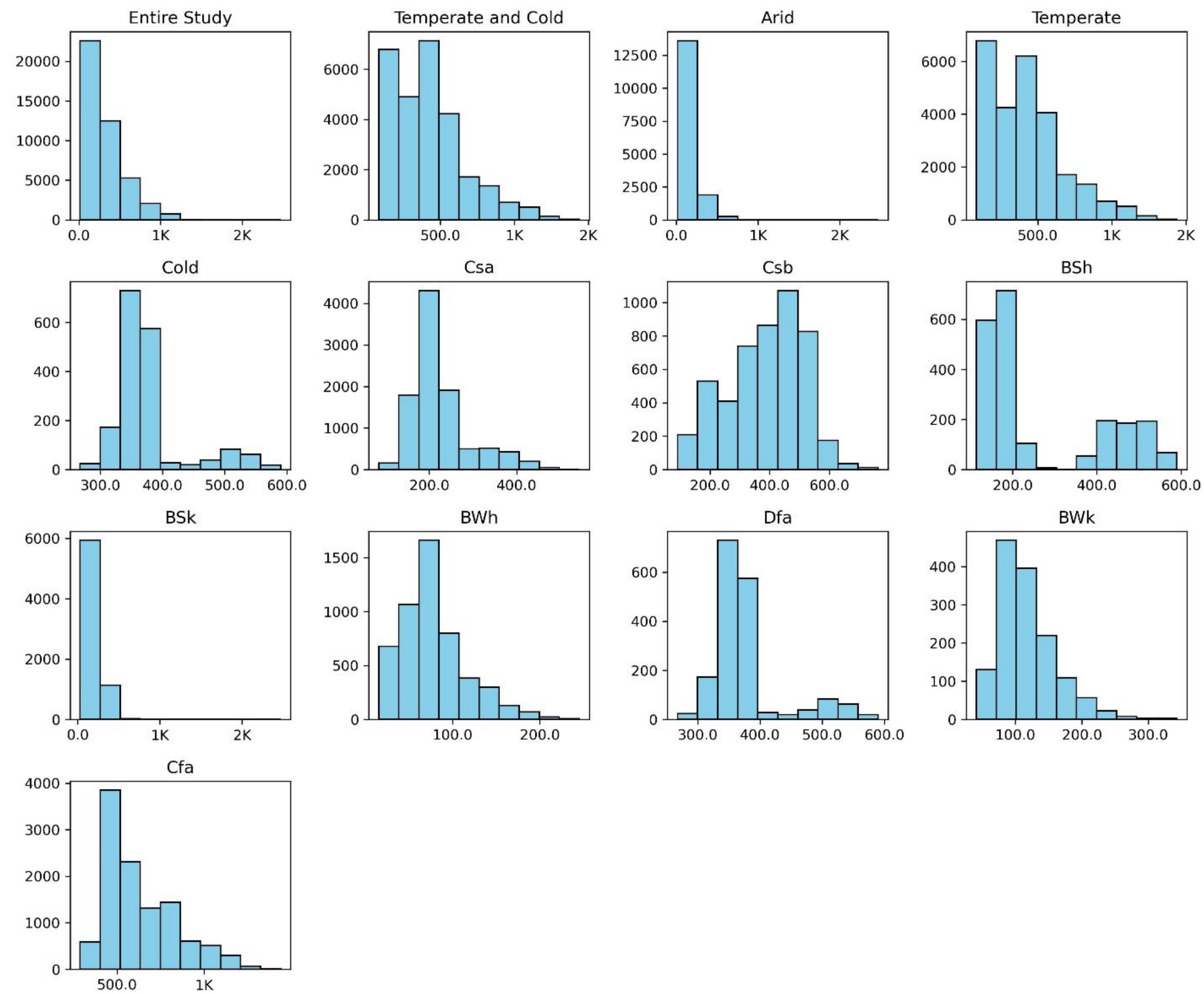

Figure S22 The distribution of actual evapotranspiration (W/m²) across spatial scales (entire study area and different climate zones).

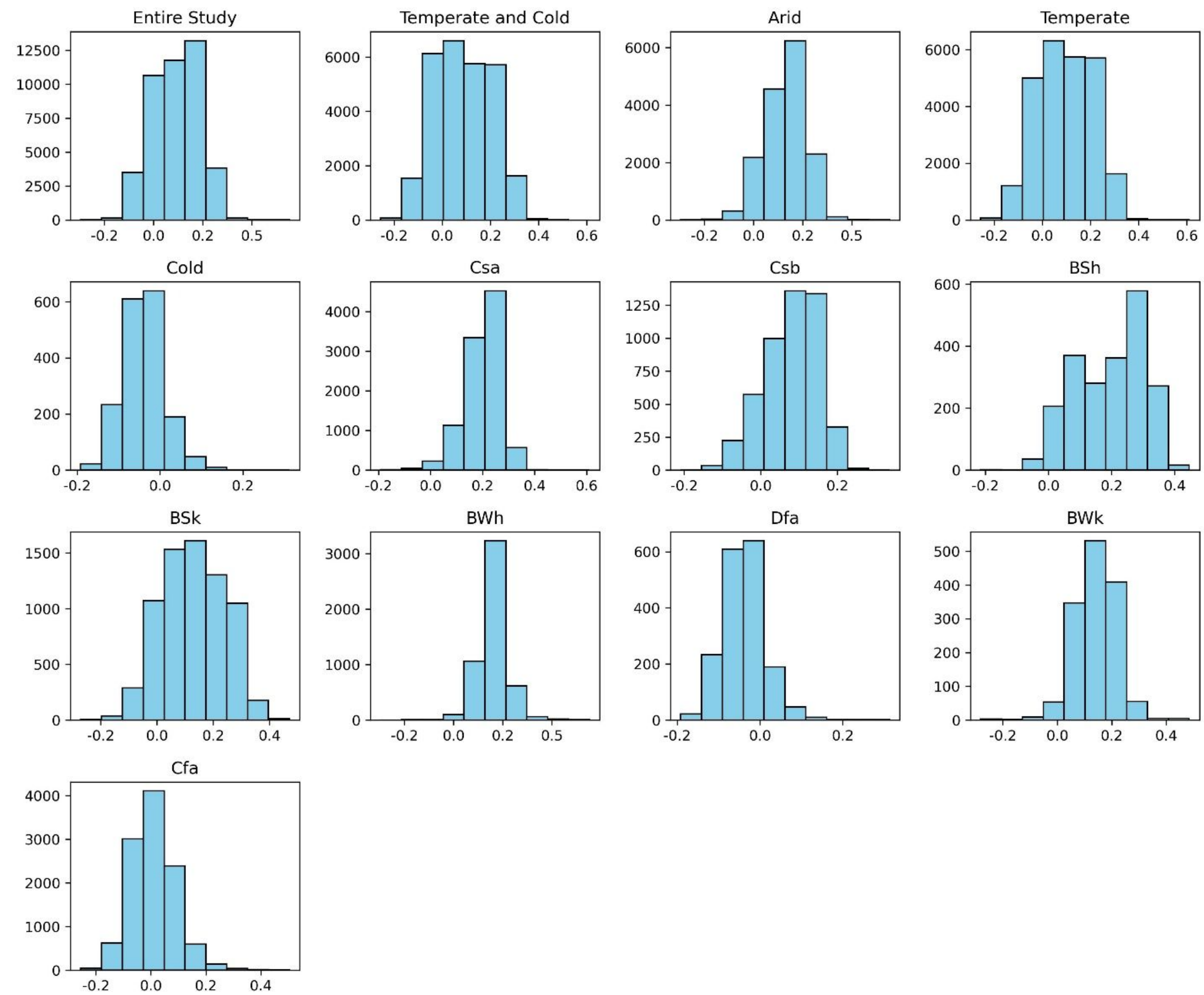

Figure S23 The distribution of land surface temperature Sen's slope (°C/year) across spatial scales (entire study area and different climate zones).

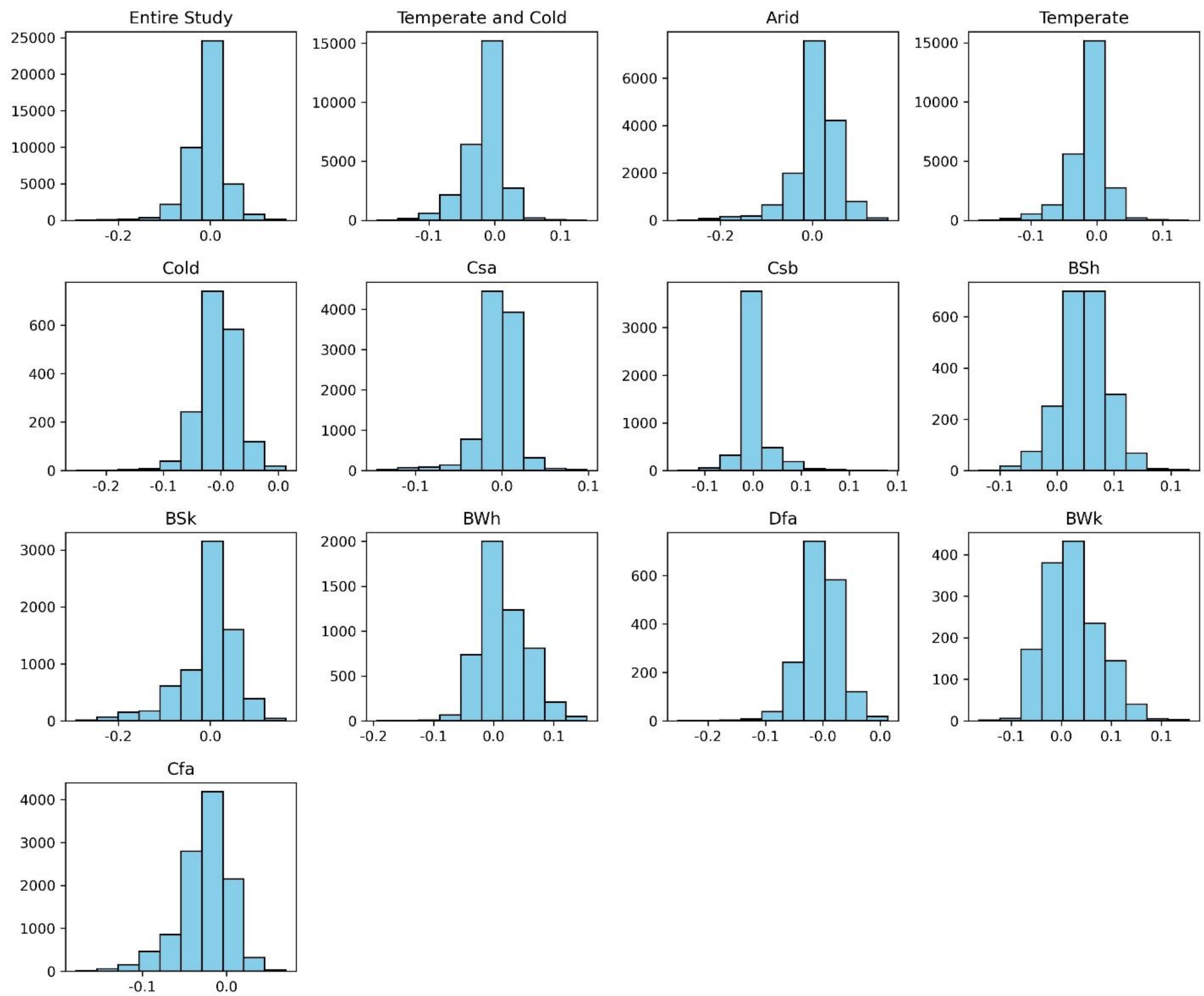

Figure S24 The distribution of actual evapotranspiration Sen's slope (mm/year) across spatial scales (entire study area and different climate zones).

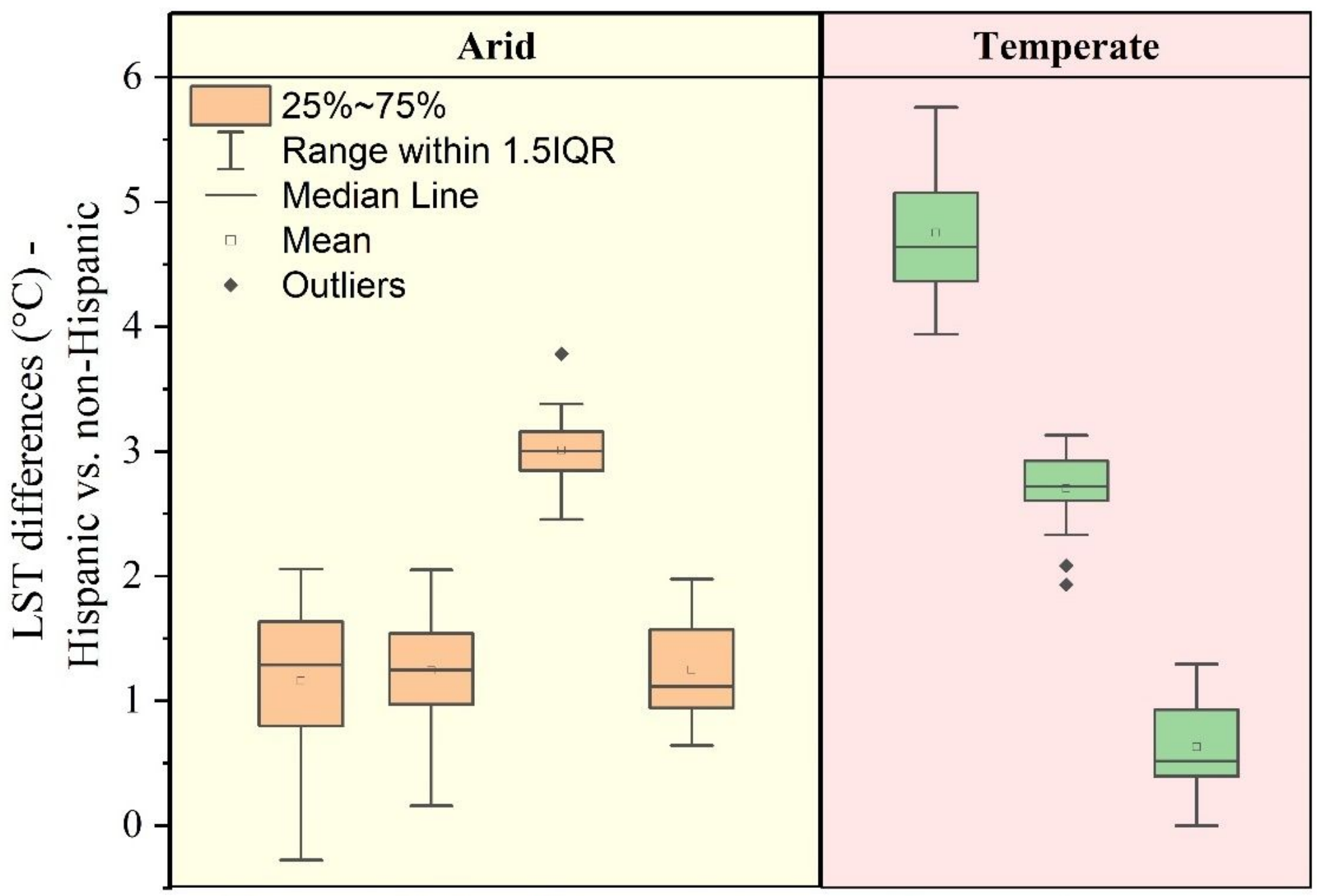

	BWk	BWh	BSh	BSk	Csa	Csb	Cfa
Mean	1.16	1.24	3.01	1.25	4.75	2.7	0.63
Median	1.29	1.25	3	1.12	4.64	2.72	0.52
Slope	0.054	0.03	0.0053	0.005	0.02	0.001	-0.0253

Figure S25 LST differences between Hispanic and non-Hispanic neighborhoods from 2000 to 2020 in connection with specific climate zones. The differences are computed by the mean LST values of Hispanic-dominated neighborhoods subtracted by the mean LST values of non-Hispanic-dominated neighborhoods. Note: <25th and >75th percentiles represent the areas with Hispanic population lower than 25th percentile (non-Hispanic-dominated neighborhoods) and higher than 75th percentile (Hispanic-dominated neighborhoods) at the city level. Climate zones: BWk-Arid, desert, hot; BWh—Arid, desert, cold; BSh—Arid, steppe, hot; BSk—Arid, steppe, cold; Csa—Temperate, dry summer, hot summer; Csb—Temperate, dry summer, warm summer; Cfa—Temperate, no dry season, hot summer; Dfa—Cold, no dry season, hot summer.

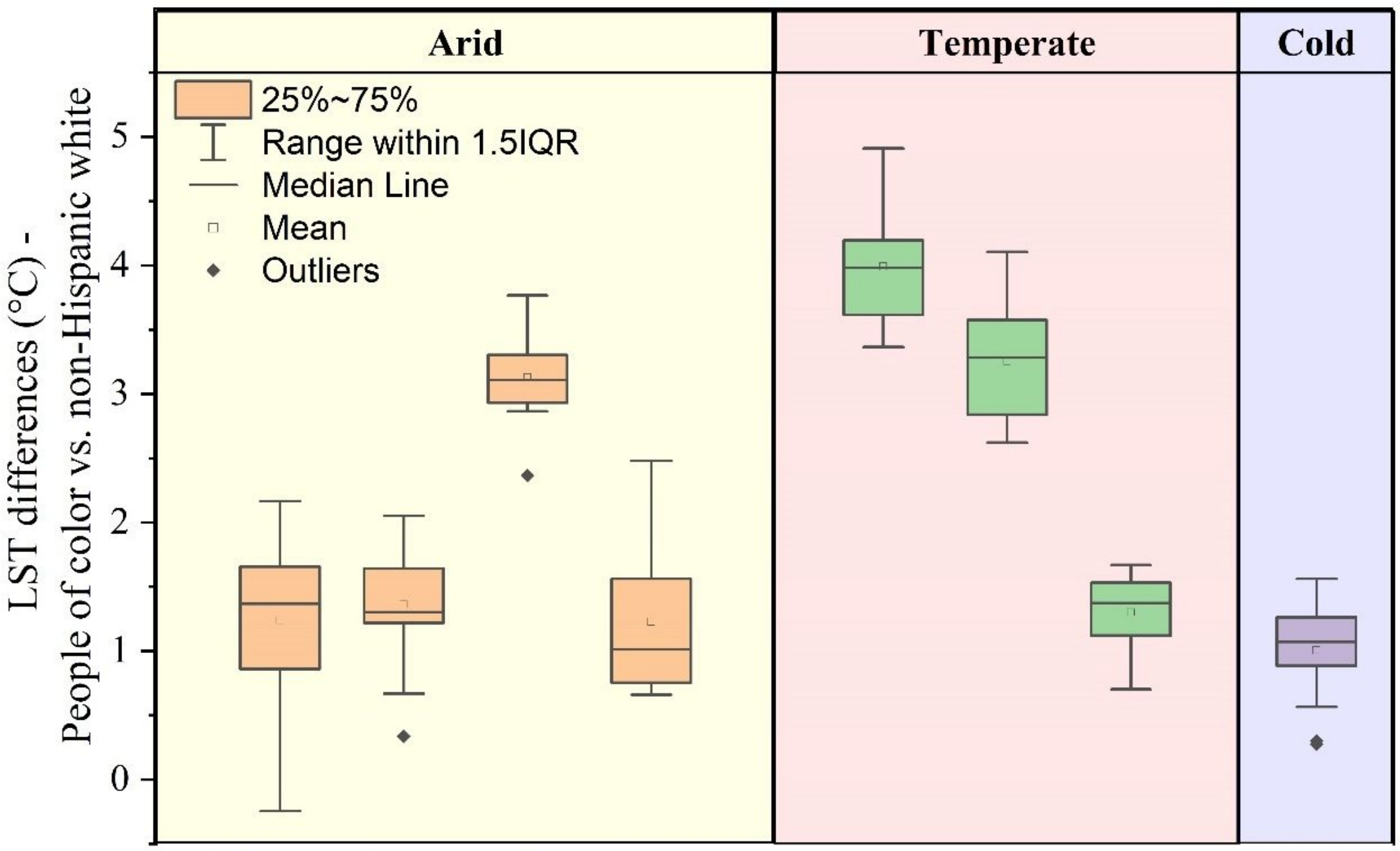

	BWh	BWk	BSh	BSk	Csa	Csb	Cfa	Dfa
Mean	1.24	1.36	3.13	1.23	3.99	3.25	1.3	1.01
Median	1.37	1.3	3.11	1.01	3.98	3.28	1.37	1.07
Slope	0.054	0.026	-0.0077	-0.023	0.013	-0.014	-0.0038	0.0067

Figure S26 LST differences between people of color and non-Hispanic White neighborhoods from 2000 to 2020 in connection with specific climate zones. The differences are computed by the mean LST values of people of color-dominated neighborhoods subtracted by the mean LST values of non-Hispanic White communities-dominated neighborhoods. Note: <25th and >75th percentiles represent the areas with people of color population lower than 25th percentile (non-Hispanic White-dominated neighborhoods) and higher than 75th percentile (people of color-dominated neighborhoods) at the city level. Climate zones: BWk-Arid, desert, hot; BWh—Arid, desert, cold; BSh—Arid, steppe, hot; BSk—Arid, steppe, cold; Csa—Temperate, dry summer, hot summer; Csb—Temperate, dry summer, warm summer; Cfa—Temperate, no dry season, hot summer; Dfa—Cold, no dry season, hot summer.

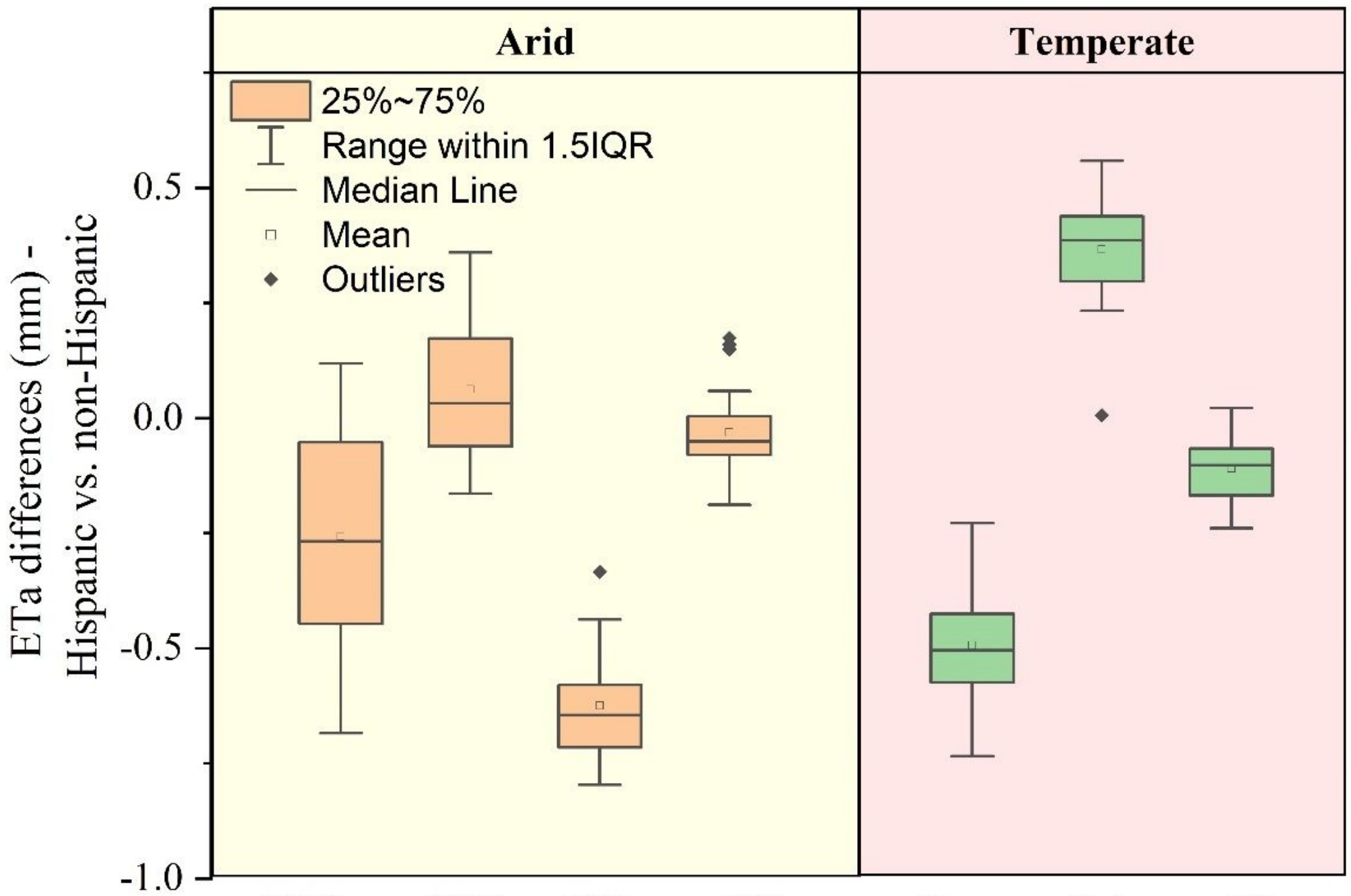

	BWk	BWh	BSh	BSk	Csa	Csb	Cfa
Mean	-0.26	0.06	-0.62	-0.03	-0.49	0.37	-0.11
Median	-0.27	0.03	-0.64	-0.05	-0.5	0.39	-0.1
Slope	-0.035	-0.016	-0.0121	0.002	0.015	0.004	-0.003

Figure S27 ETa differences between Hispanic and non-Hispanic neighborhoods from 2000 to 2020 in connection with specific climate zones. The differences are computed by the mean ETa values of Hispanic-dominated neighborhoods subtracted by the mean ETa values of non-Hispanic-dominated communities. Note: <25th and >75th percentiles represent the areas with Hispanic population lower than 25th percentile (non-Hispanic-dominated neighborhoods) and higher than 75th percentile (Hispanic-dominated neighborhoods) at the city level. Climate zones: BWk-Arid, desert, hot; BWh—Arid, desert, cold; BSh—Arid, steppe, hot; BSk—Arid, steppe, cold; Csa—Temperate, dry summer, hot summer; Csb—Temperate, dry summer, warm summer; Cfa—Temperate, no dry season, hot summer; Dfa—Cold, no dry season, hot summer.

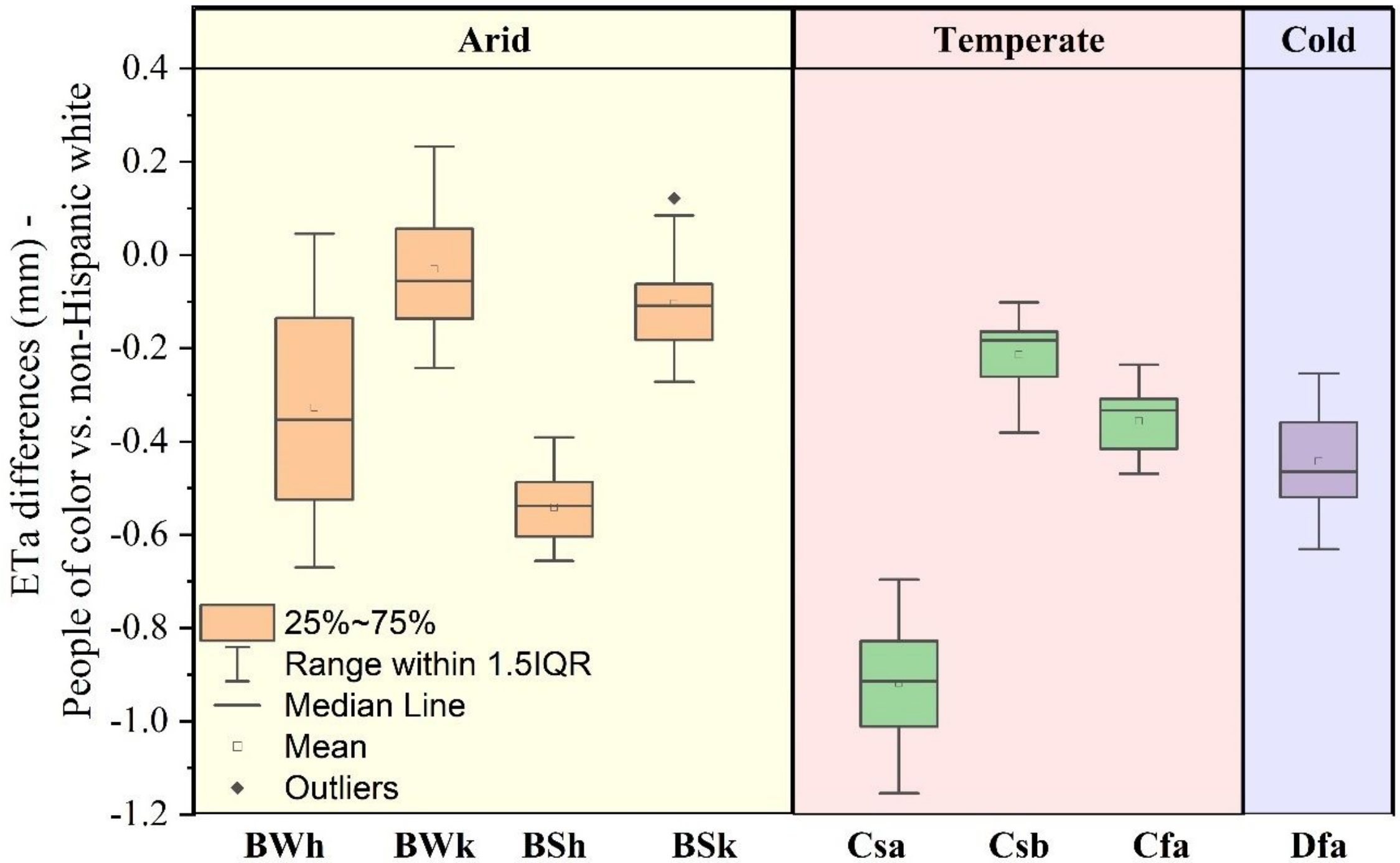

	BWh	BWk	BSh	BSk	Csa	Csb	Cfa	Dfa
Mean	-0.33	-0.03	-0.54	-0.1	-0.92	-0.21	-0.36	-0.44
Median	-0.35	-0.06	-0.54	-0.11	-0.91	-0.18	-0.33	-0.46
Slope	-0.031	-0.014	-2E-4	0.002	0.011	-0.004	-0.0058	-0.005

Figure S28 ETa differences between people of color and non-Hispanic White neighborhoods from 2000 to 2020 in connection with specific climate zones. The differences are computed by the mean ETa values of people of color-dominated neighborhoods subtracted by the mean ETa values of non-Hispanic White communities-dominated neighborhoods. Note: <25th and >75th percentiles represent the areas with people of color population lower than 25th percentile (non-Hispanic White-dominated neighborhoods) and higher than 75th percentile (people of color-dominated neighborhoods) at the city level. Climate zones: BWk-Arid, desert, hot; BWh—Arid, desert, cold; BSh—Arid, steppe, hot; BSk—Arid, steppe, cold; Csa—Temperate, dry summer, hot summer; Csb—Temperate, dry summer, warm summer; Cfa—Temperate, no dry season, hot summer; Dfa—Cold, no dry season, hot summer.

Table S1 The list of cities with total population and Köppen climate zone classification.

ID	Name	Total population	Koppen	Koppen climate zone 2
1	Tucson, AZ	868,014	Arid	BWh
2	Las Vegas--Henderson, NV	2,066,987	Arid	BWh
3	San Jose, CA	1,793,065	Temperate and Cold	Csb
4	Austin, TX	1,605,549	Temperate and Cold	Cfa
5	Lancaster--Palmdale, CA	350,923	Arid	BSk
6	Denton--Lewisville, TX	407,602	Temperate and Cold	Cfa
7	Colorado Springs, CO	608,321	Arid	BSk
8	Phoenix--Mesa, AZ	4,015,368	Arid	BWh
9	Lubbock, TX	254,921	Arid	BSk
10	Corpus Christi, TX	338,875	Temperate and Cold	Cfa
11	Dallas--Fort Worth--Arlington, TX	5,715,476	Temperate and Cold	Cfa
12	San Antonio, TX	1,983,363	Temperate and Cold	Cfa
13	Indio--Cathedral City, CA	367,875	Arid	BWh
14	Riverside--San Bernardino, CA	2,060,310	Arid	BSh
15	Houston, TX	5,594,567	Temperate and Cold	Cfa
16	Antioch, CA	306,447	Temperate and Cold	Csb
17	Stockton, CA	390,112	Temperate and Cold	Csb
18	Salt Lake City--West Valley City, UT	1,104,330	Arid	BSk
19	Provo--Orem, UT	541,045	Arid	BSk
20	Oklahoma City, OK	929,623	Temperate and Cold	Cfa
21	Laredo, TX	255,544	Arid	BSh
22	San Diego, CA	3,155,287	Arid	BSk
23	Sacramento, CA	1,838,376	Temperate and Cold	Csa
24	Santa Rosa, CA	318,780	Temperate and Cold	Csb
25	McAllen, TX	794,410	Arid	BSh
26	Murrieta--Temecula--Menifee, CA	508,003	Arid	BSh
27	Reno, NV--CA	419,627	Arid	BWk

28	Los Angeles--Long Beach--Anaheim, CA	12,563,660	Temperate and Cold	Csa
29	Conroe--The Woodlands, TX	283,143	Temperate and Cold	Cfa
30	Mission Viejo--Lake Forest--San Clemente, CA	610,051	Temperate and Cold	Csa
31	Bakersfield, CA	555,280	Arid	BWh
32	San Francisco--Oakland, CA	3,517,530	Temperate and Cold	Csb
33	Concord, CA	663,488	Temperate and Cold	Csb
34	El Paso, TX--NM	813,619	Arid	BWk
35	Modesto, CA	376,966	Arid	BSk
36	Tulsa, OK	693,588	Temperate and Cold	Cfa
37	Ogden--Layton, UT	596,032	Arid	BSk
38	Albuquerque, NM	761,430	Arid	BWk
39	Victorville--Hesperia, CA	341,039	Arid	BSk
40	Kansas City, MO--KS	1,590,067	Temperate and Cold	Dfa
41	Wichita, KS	482,017	Temperate and Cold	Dfa
42	Fort Collins, CO	295,595	Arid	BSk
43	Fresno, CA	696,171	Arid	BSk
44	Oxnard, CA	380,751	Temperate and Cold	Csb
45	Santa Clarita, CA	265,908	Temperate and Cold	Csa
46	Denver--Aurora, CO	2,643,663	Arid	BSk

Table S2 The description of data set used in this study

**Datasets**		**Resolution**	**Source/method**	**Time**
Boundary datasets	US Southwest urban areas	-	US census	2018
	US Census block groups	-	US census	2020
	Köppen-Geiger zone	1km	Download from GloH2O (http://www.gloh2o.org/koppen/)	2018
Demographic Data	Race/ethnicity	Block group level	US census	2000, 2010, 2020
Socioeconomic data	Median household income	Block group level	US census	2019
	Per capita income	Block group level	US census	2019

	Poverty status of household	Block group level	US census	2019
	Median household property values	Block group level	US census	2019
Environmental database	Daytime Land Surface Temperature	70 m	ECOSTRESS	2020
	Actual Evapotranspiration	70 m	ECOSTRESS	2020
	Evaporative Stress Index	70 m	ECOSTRESS	2020
Environmental change database	LST changes	30 m	Landsat-based Statistical Mono-Window algorithm	2000-2020
	ETa changes	30 m	Landsat-based Simplified Surface Energy Balance	2000-2020

Table S3 The list of urbanized neighborhoods was removed when we explored the inequality changes with climate changes due to only one dominated specific race/ethnicity within these neighborhoods. Suppose 75th percentile for percentage of race/ethnicity of each urban area are lower than 0.3. In that case, these neighborhoods are regarded as outliers, and we will remove these areas to compare Hispanic vs. non-Hispanic and people of color vs. non-Hispanic White.

**Category**	**Urbanized areas**
Hispanic-dominated urbanized areas	Laredo, TX; McAllen, TX; El Paso, TX--NM
Non-Hispanic-dominated urbanized areas	Kansas City, MO--KS; Fort Collins, CO; Tulsa, OK; Concord, CA; Ogden--Layton, UT; Provo--Orem, UT; Oklahoma City, OK; Mission Viejo--Lake Forest--San Clemente, CA; Colorado Springs, CO; Wichita, KS; Denton--Lewisville, TX; Sacramento, CA; Salt Lake City--West Valley City, UT; San Francisco--Oakland, CA
People of color-dominated urbanized areas	Laredo, TX; McAllen, TX; El Paso, TX—NM; Stockton, CA
Non-Hispanic White-dominated urbanized areas	Fort Collins, CO; Provo--Orem, UT; Ogden--Layton, UT

Table S4 Population Counts and Retention Rates After Exclusion Criteria by Racial/Ethnic Group

Category	Population	Population After Exclusions	Retention Percentage
Total	66,722,798		-
Hispanic	24,220,444	20,136,309	83.14%
non-Hispanic	42,502,354	30,845,251	72.57%
People of color	39,093,101	36,814,611	94.17%
non-Hispanic White	27,629,697	19,513,906	70.63%

S.1 Landsat-based LST and ETa retrieval

Landsat-based LST retrieval is computed using Statistical Mono-Window (SMW) algorithm developed by the Climate Monitoring Satellite Application Facility (CM-SAF)[1]. Besides Landsat images, the other two auxiliary datasets, atmospheric data from the National Center for Environmental Prediction (NCEP) and National Center and surface emissivity from the Advanced Spaceborne Thermal Emission and Reflection Radiometer Global Emissivity Database (ASTER GED) developed by NASA JPL, are also used to generate LST products. The SMW consists of an empirical linear relationship between LST and top-of-atmosphere (TOA) brightness temperatures in a single thermal infrared window channel[2-4]. This model is formed from a linearization of the radiative transfer equation while retaining an explicit dependency on the surface emissivity mean[5, 6]:

$$LST = A\,\frac{T_{TOA}}{\epsilon} + B\frac{1}{\varepsilon} + C \tag{1}$$

Where T_{TOA} represents the TOA brightness temperatures of a single infrared window channel. The coefficients A, B, and C are obtained from radiative transfer simulations implemented for the classes of total column water vapor (TCWV) values from the atmospheric data. E is the spectral surface emissivity calculated from the ASTER GED. More details about the SMW algorithm and the development of the GEE code are provided by Ermida et al. (2020)[7].

For Landsat-based ETa retrieval, the operational version of Simplified Surface Energy Balance (SSEBop) is used to construct ETa maps of the study area. The framework of SSEBop can provide accurate and repeatable estimates of ETa by combination with remote-sensing technology, surface energy balance methods, and local weather datasets[8]. The estimation of ETa (actual ET) with

SSEBop is performed by calculating two critical parameters, ET fraction (ET_f) and ETo using the following Equation[9]:

$$ET_a = ET_f \times kET_o (2)$$

where ETo is the grass-reference potential ET (mm), which refers to water transpired by grass with never short of water. *ETo* can be calculated by National Centers for Environmental Prediction (NCEP) Climate Forecast System (CFS) with the standardized Penman-Monteith Equation[10]. *k* is a scaling coefficient, ranging from 1.00 to 1.25[11, 12], which scales up the grass-reference ETo to maximum ETa derived from an alfalfa reference. We select k values of 1.225[11, 12]. ET_f is then determined using the following Equation[8]:

$$ET_f = \frac{T_h - T_s}{T_h - T_c} (3)$$

where T_s is the land surface temperature (LST) calculated with the Landsat thermal band; T_h and T_c represent the estimated T_s at the idealized reference "hot" and "cold," respectively. The difference between T_h and T_c can be represented as *dT*, which can be obtained using the net radiation R_n over dry-bare soil.

More details regarding the ETa calculation can be referenced by Senay et al. (2013, 2016, 2020)[8, 9, 12]. The script for the GEE platform was developed applying the SSEBop model to estimate ETa at 30 m resolution according to Senay et al., [8, 9, 12] and Lima (2019)[13]. We obtained Landsat series (TM, ETM +, and OLI / TIRS), NCEP/CFSv2 grid weather data, and digital elevation model (DEM) ETOPO1 to estimate ETa in US Southwest urban areas. The space distribution diagram of ECOSTRESS-based environmental (2020), and Landsat-based environmental change (2000–

2020) metrics over the state capitals within our study areas are shown in Figures S2-S6. All datasets can be accessed via Zenodo at http://tinyurl.com/bdsdzj8y.

S.2 Inequality changes with climate change

As shown in Tables 1–2, the surrounding environment deteriorated more rapidly for racial and ethnic minorities than for non-Hispanic and non-Hispanic White during the study period. We observed greater differences in LST and ETa changes between ethnic groups than between racial groups. Distinct differences in heat exposure between the contrary groups, measured by LST, were observed in the study area from 2000 to 2020 (Figure S16). The LSTs in Hispanic-/people of color-dominated neighborhoods (>75th) were consistently the highest, followed by those of the ethnically/racially diverse neighborhoods (25th–75th), while non-Hispanic-/non-Hispanic White-dominated neighborhoods (<25th) experienced only modest urban warming effects (Figure S16a and S16b). For ETa changes, non-Hispanic-dominated neighborhoods showed ETa values similar to those of Hispanic-dominated neighborhoods, and ethnically diverse neighborhoods had the highest ETa values over the 21 years surveyed (Figure S16c). Such results are beyond expectations and are different from the results of people of color against non-Hispanic Whites. ETa values of different areas presented notable distinctions, with the highest values in non-Hispanic White-dominated neighborhoods, followed by racially diverse neighborhoods, and the worst ETa conditions in people of color-dominated neighborhoods during 2000–2020 (Figure S16d).

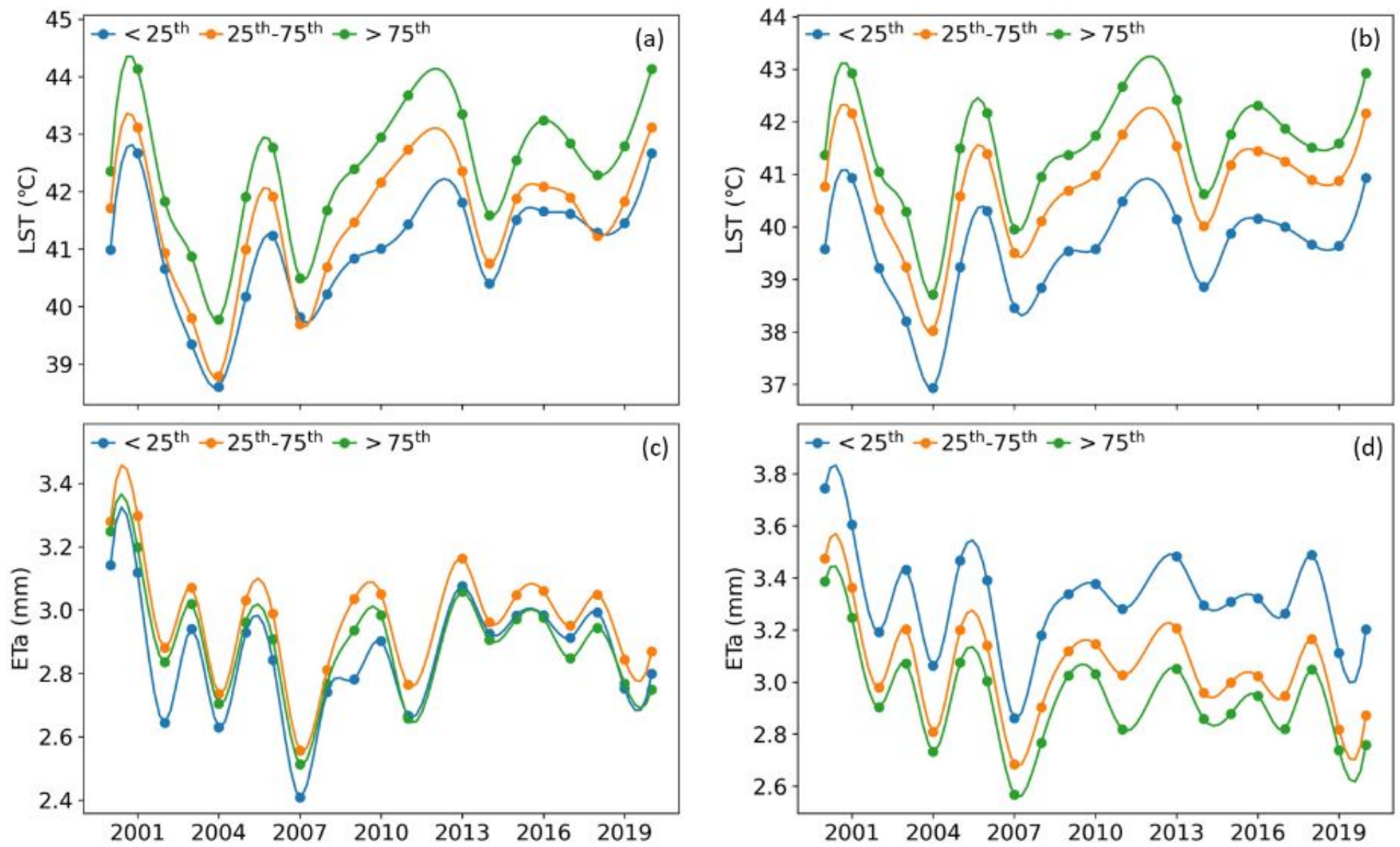

Figure S29 The time series of LST and ETa annual means from 2000 to 2020 of contrary groups: (a) LST changes for Hispanic versus non-Hispanic, (b) LST changes for people of color versus non-Hispanic White, (c) ETa changes for Hispanic versus non-Hispanic, and (d) ETa changes for people of color versus non-Hispanic White. Note: <25th, 25th – 75th, and >75th percentiles represent the areas with a population of Hispanic/people of color lower than 25th (non-Hispanic-/non-Hispanic White-dominated neighborhoods), between 25th and 75th (ethnically/racially diverse neighborhoods), and higher than 75th percentiles (Hispanic-/people of color-dominated neighborhoods) at the city level, respectively.

Unprecedented and accelerating global climate change has the potential to impact environmental justice [14, 15]. The Intergovernmental Panel on Climate Change has reported climate change's impacts, adaptation, and vulnerability [16]. However, evidence-based studies on the modulation of inequalities between groups of people by climate change remain scarce. Given the potential for the complex dynamics of environmental factors, we illustrate the interactions between inequalities and environmental changes (LST and ETa changes). Overall, LST showed a remarkably increasing trend, and ETa presented a decreasing trend in the context of global warming and drought conditions across the US Southwest, implying that the study areas have become hotter and drier during the 21 years surveyed. These findings are consistent with prior research reporting the intensification of hot and dry extremes in the region [17-21]. Accordingly, all racial/ethnic groups

experienced positive LST trends and negative ETa trends, though the magnitude of these changes varied across groups.

References:

(1) Duguay-Tetzlaff, A.; Bento, V. A.; Göttsche, F. M.; Stöckli, R.; Martins, J.; Trigo, I.; Olesen, F.; Bojanowski, J. S.; Da Camara, C.; Kunz, H. Meteosat land surface temperature climate data record: Achievable accuracy and potential uncertainties. *Remote Sensing* **2015**, *7* (10), 13139-13156.

(2) Freitas, S. C.; Trigo, I. F.; Macedo, J.; Barroso, C.; Silva, R.; Perdigão, R. Land surface temperature from multiple geostationary satellites. *International Journal of Remote Sensing* **2013**, *34* (9-10), 3051-3068.

(3) Li, Z.-L.; Tang, B.-H.; Wu, H.; Ren, H.; Yan, G.; Wan, Z.; Trigo, I. F.; Sobrino, J. A. Satellite-derived land surface temperature: Current status and perspectives. *Remote sensing of environment* **2013**, *131*, 14-37.

(4) Sun, D.; Pinker, R. T.; Basara, J. B. Land surface temperature estimation from the next generation of Geostationary Operational Environmental Satellites: GOES M–Q. *Journal of Applied Meteorology and Climatology* **2004**, *43* (2), 363-372.

(5) Jesdale, B. M.; Morello-Frosch, R.; Cushing, L. The racial/ethnic distribution of heat risk–related land cover in relation to residential segregation. *Environmental health perspectives* **2013**, *121* (7), 811-817.

(6) Morello-Frosch, R.; Lopez, R. The riskscape and the color line: examining the role of segregation in environmental health disparities. *Environmental research* **2006**, *102* (2), 181-196.

(7) Ermida, S. L.; Soares, P.; Mantas, V.; Göttsche, F.-M.; Trigo, I. F. Google earth engine open-source code for land surface temperature estimation from the landsat series. *Remote Sensing* **2020**, *12* (9), 1471.

(8) Senay, G. B.; Bohms, S.; Singh, R. K.; Gowda, P. H.; Velpuri, N. M.; Alemu, H.; Verdin, J. P. Operational Evapotranspiration Mapping Using Remote Sensing and Weather Datasets: A New Parameterization for the SSEB Approach. *Journal of the American Water Resources Association* **2013**, *49* (3), 577-591. DOI: 10.1111/jawr.12057.

(9) Senay, G. B.; Kagone, S.; Velpuri, N. M. Operational global actual evapotranspiration: Development, evaluation, and dissemination. *Sensors (Switzerland)* **2020**, *20* (7), 1-18. DOI: 10.3390/s20071915.

(10) Allen, R. G.; Pereira, L. S.; Raes, D.; Smith, M. Crop evapotranspiration-Guidelines for computing crop water requirements-FAO Irrigation and drainage paper 56. *Fao, Rome* **1998**, *300* (9), D05109.

(11) de Andrade, B. C. C.; de Andrade Pinto, E. J.; Ruhoff, A.; Senay, G. B. Remote sensing-based actual evapotranspiration assessment in a data-scarce area of Brazil: A case study of the Urucuia Aquifer System. *International Journal of Applied Earth Observation and Geoinformation* **2021**, *98*, 102298. DOI: https://doi.org/10.1016/j.jag.2021.102298.

(12) Senay, G. B.; Friedrichs, M.; Singh, R. K.; Velpuri, N. M. Evaluating Landsat 8 evapotranspiration for water use mapping in the Colorado River Basin. *Remote Sensing of Environment* **2016**, *185*, 171-185.

(13) Lima, R. N. d. S. *SSEBop GEE for Landsat Time Series*. 2019. https://zenodo.org/record/3620925#.YifPROjMKzU (accessed 2022 2022-03-08).

(14) Kasperson, J. X.; Kasperson, R. E.; Turner, B.; Hsieh, W.; Schiller, A. Vulnerability to global environmental change. In *The social contours of risk*, Routledge, 2022; pp 245-285.

(15) Jbaily, A.; Zhou, X.; Liu, J.; Lee, T.-H.; Kamareddine, L.; Verguet, S.; Dominici, F. Air pollution exposure disparities across US population and income groups. *Nature* **2022**, *601* (7892), 228-233. DOI: 10.1038/s41586-021-04190-y.

(16) Pörtner, H.-O.; Roberts, D. C.; Adams, H.; Adler, C.; Aldunce, P.; Ali, E.; Begum, R. A.; Betts, R.; Kerr, R. B.; Biesbroek, R. *Climate change 2022: Impacts, adaptation and vulnerability*; IPCC Geneva, Switzerland:, 2022.

(17) McKinnon, K. A.; Poppick, A.; Simpson, I. R. Hot extremes have become drier in the United States Southwest. *Nature Climate Change* **2021**, *11* (7), 598-604.

(18) Lund, J.; Medellin-Azuara, J.; Durand, J.; Stone, K. Lessons from California's 2012–2016 drought. *Journal of Water Resources Planning and Management* **2018**, *144* (10), 04018067.

(19) Robeson, S. M. Revisiting the recent California drought as an extreme value. *Geophysical Research Letters* **2015**, *42* (16), 6771-6779.

(20) Ullrich, P.; Xu, Z.; Rhoades, A.; Dettinger, M.; Mount, J.; Jones, A.; Vahmani, P. California's drought of the future: A midcentury recreation of the exceptional conditions of 2012–2017. *Earth's Future* **2018**, *6* (11), 1568-1587.

(21) Demetillo, M. A. G.; Anderson, J. F.; Geddes, J. A.; Yang, X.; Najacht, E. Y.; Herrera, S. A.; Kabasares, K. M.; Kotsakis, A. E.; Lerdau, M. T.; Pusede, S. E. Observing severe drought influences on ozone air pollution in California. *Environmental science & technology* **2019**, *53* (9), 4695-4706.
